# Intercomparison of Unmanned Aerial Vehicle and Ground-Based Narrow Band Spectrometers Applied to Crop Trait Monitoring in Organic Potato Production

**DOI:** 10.3390/s17061428

**Published:** 2017-06-18

**Authors:** Marston Héracles Domingues Franceschini, Harm Bartholomeus, Dirk van Apeldoorn, Juha Suomalainen, Lammert Kooistra

**Affiliations:** 1 Laboratory of Geo-Information Science and Remote Sensing, Wageningen University and Research, P.O. Box 47, 6700 AA Wageningen, The Netherlands; harm.bartholomeus@wur.nl (H.B.); juha.suomalainen@nls.fi (J.S.); lammert.kooistra@wur.nl (L.K.); 2 Farming Systems Ecology Group, Wageningen University and Research, P.O. Box 430, 6700 AK Wageningen, The Netherlands; dirk.vanapeldoorn@wur.nl; 3 Finnish Geospatial Research Institute, National Land Survey of Finland, Geodeetinrinne 1, 02430 Masala, Finland

**Keywords:** hyperspectral imagery, Vis-NIR spectroscopy, organic cropping systems, vegetation indices

## Abstract

Vegetation properties can be estimated using optical sensors, acquiring data on board of different platforms. For instance, ground-based and Unmanned Aerial Vehicle (UAV)-borne spectrometers can measure reflectance in narrow spectral bands, while different modelling approaches, like regressions fitted to vegetation indices, can relate spectra with crop traits. Although monitoring frameworks using multiple sensors can be more flexible, they may result in higher inaccuracy due to differences related to the sensors characteristics, which can affect information sampling. Also organic production systems can benefit from continuous monitoring focusing on crop management and stress detection, but few studies have evaluated applications with this objective. In this study, ground-based and UAV spectrometers were compared in the context of organic potato cultivation. Relatively accurate estimates were obtained for leaf chlorophyll (RMSE = 6.07 µg·cm^−2^), leaf area index (RMSE = 0.67 m^2^·m^−2^), canopy chlorophyll (RMSE = 0.24 g·m^−2^) and ground cover (RMSE = 5.5%) using five UAV-based data acquisitions, from 43 to 99 days after planting. These retrievals are slightly better than those derived from ground-based measurements (RMSE = 7.25 µg·cm^−2^, 0.85 m^2^·m^−2^, 0.28 g·m^−2^ and 6.8%, respectively), for the same period. Excluding observations corresponding to the first acquisition increased retrieval accuracy and made outputs more comparable between sensors, due to relatively low vegetation cover on this date. Intercomparison of vegetation indices indicated that indices based on the contrast between spectral bands in the visible and near-infrared, like OSAVI, MCARI2 and CI_g_ provided, at certain extent, robust outputs that could be transferred between sensors. Information sampling at plot level by both sensing solutions resulted in comparable discriminative potential concerning advanced stages of late blight incidence. These results indicate that optical sensors, and their integration, have great potential for monitoring this specific organic cropping system.

## 1. Introduction

In-season monitoring of crop development can assist management practices in different aspects, allowing farmers to optimize agricultural operations according to site-specific characteristics [1]. Information obtained in previous seasons can also be considered for planning the cultivation of subsequent crops, taking into account the spatiotemporal variability of yield and of different factors related to the production [1,2]. In parallel, high-throughput approaches for field-based phenotyping can assist plant breeding in order to identify genotypes with better performance under diverse abiotic and biotic constraints, under realistic production conditions [3]. In these cases, proximal and remote sensing offers a multitude of techniques that can be applied to estimate crop traits over time in non-destructive and spatially explicit ways. For instance, reflectance in the optical domain has been used to retrieve plants biochemical and biophysical properties, with high potential for the assessment of leaf pigments, like chlorophylls, and leaf area index, amongst other traits of great importance in the evaluation of plant health and of its response to stress conditions [4,5].

The estimation of leaf pigments content and canopy structural traits through optical remote sensing, usually with measurements of reflectance in spectral bands between 450 and 900 nm (i.e., from visible to near-infrared), is performed using approaches that can be broadly divided in three categories [6]: empirical-statistical (i.e., through parametrical or non-parametrical regressions), physical-based and hybrid. The first group comprises calculations of vegetation indices, which are derived considering statistical or physical relations between vegetation properties and its spectral response, and also statistical-based methods, which rely on “best-fit” or regression optimization for a given training dataset [7]. Physical methods, on the other hand, are based on detailed mathematical description of the interactions between incident energy with vegetation and background (i.e., soil), considering the major factors related to this relationship (e.g., view and illumination geometry, leaf and canopy characteristics, etc.), through radiative transfer models (RTMs). Although physical approaches have a greater generalization potential their parametrization and inversion are considerably more complex and require substantial effort to be implemented in practical frameworks. In order to mitigate these limitations, RTMs can be inverted using non-parametric regressions trained upon simulated data originating hybrid methods, which give a simplified inversion alternative to physical approaches. Despite the fast development of more advanced retrieval techniques based on the physical description of crop spectral response, vegetation indices still are a valuable alternative to perform plat traits modeling in a straightforward and robust way with applications in the assessment of crop health and phenomics [8,9,10].

The timely acquisition of spectral information is a key factor for crop monitoring during the growing season concerning the detection of stress factors and assessment of their impact on the crop development [1]. To that end, integration of data streams from different optical sensors can bring flexibility to follow crop growth over time, since acquisition can be performed using different platforms, according to environmental constraints, and measurements may be potentially coupled with other agricultural operations [4]. In this respect, simultaneous use of point-based data or images acquired using terrestrial sensors with spatially continuous information derived from UAV, airborne or satellite systems can provide a more suitable monitoring framework than relying on a single information source, especially considering temporal resolution demanded by agricultural applications and phenotyping methodologies [11,12]. 

Multiple approaches are available to integrate datasets from optical sensors. For instance, intercalibration methods are generally applied to combine reflectance or vegetation indices derived from different satellite systems [13,14,15,16], although its application to sensors in other platforms has been evaluated as well [17,18]. In this context, the use of vegetation indices may simplify the transfer procedure since they are designed to mitigate background effects and changes in illumination conditions over time. Also, RTMs might be included in frameworks intending to extend the analysis to outputs of different sensors, considering the generalization potential of these methods [6]. However, any integration approach will be potentially subject to bias introduced by the sensors and data acquisition characteristics (e.g., spectral response, data spatial support, information sampling scheme) and the interaction of these factors with illumination conditions and sun-target-sensor geometry. Therefore evaluating the impact that using different sensors, at different acquisition levels, has upon crop traits retrieval and plant development monitoring may help to indicate limitations and potentials of integrate sensing frameworks applied to the context of crop phenotyping or general vegetation growth monitoring.

The retrieval of crop traits during the growing season can be particularly challenging for organic cropping systems. For instance, in organic production of potato (*Solanum tuberosum*), nitrogen and late blight (*Phytophthora infestans*) are in general the most important aspects affecting the crop development, due to the constraints on the use of chemical fertilizers and synthetic pesticides [19]. Nutrient shortage and pathogen development can occur simultaneously with other stress factors, increasing the complexity of the system. In addition, the estimation of vegetation properties can be particularly challenging in initial stages of the crop development while canopy is still largely open and considerable amount of background can affect the signal measured by a given sensor [20]. These factors, together with potential variations in the illumination condition and sun-target-sensor geometry between different data acquisitions [21], increase the difficulty of crops traits retrieval. Despite that, continuous monitoring of crop development in organic production can be useful to stablish management according to the variability observed in the field, concerning, for example, fertilization and varietal choices. However, research focusing on the application of precision agriculture practices in organic cropping is still scarce [22], and solutions suitable to this particular cultivation system need to be established.

Therefore, the present study has the following objectives: (a) evaluate the accuracy of simple retrieval methods (i.e., calculation of vegetation indices from narrow band spectral data coupled with linear regression) for predicting organic potato traits during the growing season, based on UAV hyperspectral data; (b) compare results obtained using UAV imagery with those derived from ground-based measurements concerning crop traits estimation and discriminative potential of the information derived, in this case focusing on late blight development; (c) intercompare data derived from both acquisition levels (i.e., UAV- and ground-based) in order to evaluate implications that a potential integration of different sensing approaches may have in agricultural applications or phenotyping frameworks.

## 2. Materials and Methods 

### 2.1. Study Site 

**Figure 1:**
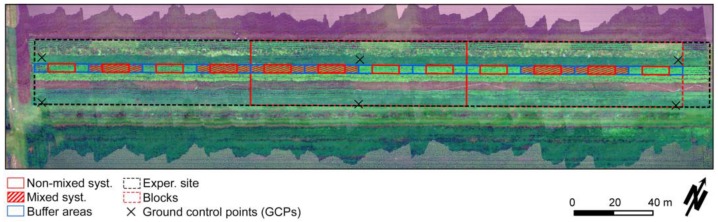
Location of the plots in the experimental area, represented over true color composite of a hyperspectral image, acquired in 24 June 2015. Bands centered at 665, 555 and 485 nm are used in the RGB representation, respectively.

The dataset used in this study was acquired on twelve plots cultivated with potato, during the 2015 growing season. These plots were located within an stripcropping experiment (51.9917° N, 5.66332° E; WGS84) started in 2014, at the Droevendaal experimental farm of Wageningen University in The Netherlands. As illustrated in Figure 1, plots were arranged in a strip along the experimental site and measured 3 m × 10 m. Buffer areas, measuring 3 m × 5 m, were placed before and after each plot, in the same strip, in order to separate them and avoid border effects along the experiment. The crop was planted on 23 April 2015, with 0.75 m of inter-row spacing and 0.30 m of interplant distance, and was harvested on 10 August of the same year. Two different cultivation methods were compared: (a) plots in which a variety mixture of four different cultivars, “Annabelle”, “Ditta”, “Tiamo” and “Toluca”, with different degrees of resistance (from low to high, respectively) to late blight, were iterated in each crop row, referred as mixed crop system; and (b) plots cultivated with only one potato variety (“Toluca”) considered highly resistant to late blight, named non-mixed system. The experiment followed a generalized randomized block design, with 3 blocks and 2 replicates of each treatment (i.e., cultivation methods) in each block (Figure 1). The main objective of this study was to evaluate crop development and disease occurrence for these two organic production systems. The potential of ground-based and UAV-carried sensors for monitoring plant growth over time was tested in this context. For that, late blight severity was assessed at the plot level in different dates, as indicated in Section 2.2 (Table 1), according to the methodology described by the European and Mediterranean Plant Protection Organization (EPPO) [23].

### 2.2. UAV-Based Hyperspectral Data Acquisition and Processing

Hyperspectral data was acquired during the growing season using the WageningenUR Hyperspectral Mapping System (HYMSY), an UAV-based pushbroom imaging system [24]. It comprises a custom spectrometer (PhotonFocus SM2-D1312 camera – PhotonFocus AG, Lachen, SZ, Switzerland – with a Specim ImSpector V10 2/3 spectrograph – Specim, Spectral Imaging Ltd., Oulu, Finland), a photogrammetric camera (Panasonic GX1 16 MP – Panasonic Corp., Osaka, Japan – with 14 mm pancake lens), an integrated GPS and inertial navigation system (INS) unit (XSens MTi-G-700 – Xsens Technologies BV, Enschede, The Netherlands), together with synchronization and data sink elements. The equipment weights approximately 2 kg, allowing its use with relatively small-size UAVs resulting in a flexible hyperspectral sensing platform for in season vegetation monitoring.

Digital numbers (DN) obtained with the UAV-based spectrometer were converted to radiance units using dark current and flat field calibration. Wavelengths were standardized to allow comparison over the complete sensor field-of-view yielding a resampled radiance data in the interval between 450 and 915 nm, with 10 nm spectral resolution. A 25% reflectance spectralon panel was measured before each flight as reference target to convert the radiances to reflectance factors considering illumination conditions.

The geometric correction of the hyperspectral datacube acquired was performed through a direct georeferencing procedure, which requires an external digital surface model (DSM) and GPS-INS data with high accuracy [25]. In the case of the HYSMY, a DSM was obtained from images taken with the photogrammetric camera included in the system, using structure-from-motion algorithm [26], implemented through the photogrammetric software PhotoScan Pro v1.0.0 (Agisoft LLC, St. Petersburg, Russia). At the same time that the high resolution DSM was derived, adjusted camera orientations that matched the surface model were obtained. These adjusted orientations were used to increase accuracy of the GPS-INS data before geometric correction of the hyperspectral data, which is a necessary step considering the relative inaccuracy of the miniature GPS-INS unit included in the system.

The resulting DSM and updated GPS-INS data were feed together with the hyperspectral datacube to the PARGE algorithm (v3.2beta, ReSe Applications, Schläpfer et al. [25]) in order to implement the georeferencing of the hyperspectral datacube. Considering that a conventional GPS receiver was used during the flights, a final georeferencing step was necessary to increase positioning accuracy not only for the hyperspectral datacube, but also for the DSM and photogrammetric orthomosaic. Therefore, a first order polynomial transformation was fitted based on six ground control points (GCPs, Figure 1), which were registered using a RTK-GPS system.

Images were acquired on five different days during the growing season, corresponding to distinct crop growth stages, as indicated in Table 1. During data acquisition the sensor system was positioned at 80 m altitude resulting in an approximate spatial resolution of 0.2 m for the hyperspectral imagery and of 0.02 m for the RGB orthomosaics. DSM with the same spatial resolution of hyperspectral datacube was derived from the orthoimages to be used in the geometric correction of the hyperspectral data.

### 2.3. Spectra Extraction from Hyperspectral Images 

Residual geometric distortion could be observed in the hyperspectral imagery even after applying the data processing methods described in Section 2.2, especially different degrees of “roll-related” bias (i.e., errors across the flight direction). This can be attributed to the interpolation performed while deriving the final orientations for the hyperspectral data [24], and the distortion intensity is probably related to flight conditions (i.e., wind speed and its variation at the flight altitude). The advantage of the geometric correction adopted here is that it depends only on in-flight acquired data, allowing a predominantly automated post-acquisition data processing procedure. Other authors proposed alternative approaches to increase geometric correction accuracy for hyperspectral datacubes obtained from UAV-based pushbroom systems, for example, relying on GCPs, tie points and linear features identified in high resolution images from RGB frame cameras [29]. However, these methods are generally more complex and require additional steps besides the flight operation itself.

Therefore, to minimize the impact of geometric inaccuracies in the final dataset to be analyzed, spectral information at plot level was extracted based on plot boundaries identified in the hyperspectral imagery using a straightforward edge-detection approach [30], implemented over values of vegetation indices. This method depended on contrasting intensities for vegetation indices corresponding to crops in adjacent strips in the experiment and between soil and vegetation in the space between strips. Different vegetation indices were used (i.e., NDVI, REP, WDVI and TCARI/OSAVI; Table 2) and results yielding the sharpest plot boundaries for each acquisition date were retained. As parameters for the Canny edge-detection algorithm the sigma value, which determines the width of the Gaussian filter applied to the image before segmentation, was set to 0.5 and the maximum and minimum thresholds adopted were the average of the directional gradient intensities for a given image and 40% of this value, respectively. Images corresponding to each acquisition were divided in four segments for processing, and calculations were performed for each segment separately.

Although the decision about the final boundaries was made visually, adding bias to the process, this simple approach helped to mitigate georeferencing errors across the flight line in the selection of the hyperspectral data. Errors along the flight direction were not considered, i.e., plot limits were assumed to be the same as those located in the field using a RTK-GPS, since the buffer areas before and after each plot received the same treatment as the corresponding plot and residual geometric distortions were estimated to be considerable lower than the dimensions of these buffer areas. Therefore any signal mixing between spectra corresponding to plots and buffer areas would probably result in low impact in the final information extracted.

### 2.4. Ground-Based Spectral Measurements

**Figure 2:**
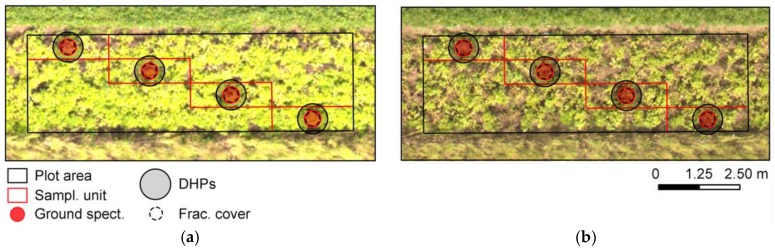
Sampling scheme at plot level represented over RGB orthomosaic for one plot cultivated with the non-mixed treatment (**a**) and other planted with mixed varieties (**b**), on the last acquisition date (31 July 2015). Transparent red circles represent ground-based spectrometer footprint (i.e., measurement area at the top of the canopy) while red rectangles indicate sampling units, in which chlorophyll measurements were made (as described in Section 2.5). Digital hemispherical photographs (DHPs) were taken pointing to the center of the sampling units, and the area sampled by pictures is indicated using transparent black circles (as described in Section 2.6). Small circles indicated by dashed black lines correspond to the area considered to derive fractional vegetation cover from DHPs (as described in Section 2.6).

Following UAV data acquisitions, canopy reflectance was measured using a handheld Cropscan Multispectral Radiometer (MSR16R; CROPSCAN Inc., Rochester, MN, USA), which performs readings in 16 spectral bands in the visible, near-infrared and shortwave infrared (from 490 to 1650 nm). Only data until 870 nm was used for analysis resulting in reflectance values for eleven bands with FWHM (full width at half maximum) varying between 7.3 and 13 nm. Measurements were made at 1 m high from the top of the crop canopy, and considering the sensor field-of-view (28°) the effective measured area was equivalent to 0.20 m^2^. More details about the sensor and data acquisition procedures are described by Clevers and Kooistra [4]. The reflectance derived using this sensor was calculated based on simultaneous measurements of down-welling irradiance, using a cosine-correct diffusor, and up-welling radiance. Spectral acquisition followed a consistent intra-plot sampling scheme, with readings recorded in four distinct points, according to a diagonal arrangement along each plot (Figure 2). One measurement was acquired per crop row in a regularly spaced pattern, which was retrieved during data acquisition using a measuring tape extended from the plot borders. Therefore, during each data acquisition 48 spectral measurements were obtained over the crop strip and spectra was averaged at plot level (i.e., the average reflectance was calculated bandwise, considering the four spectral measurements acquired in each plot) resulting in 12 spectral signatures per acquisition date, after aggregation.

### 2.5. Chlorophyll Content at Leaf and Canopy Levels

Leaf chlorophyll content was determined based on SPAD meter readings (SPAD-502; Minolta Corporation Ltd., Osaka, Japan). This sensor derive a measure based on the transmittance in red (650 nm) and near infrared (940 nm) wavelengths [31]. Data collection followed the same intra-plot sampling scheme described in Section 2.3, and, therefore, measurements were recorded in four distinct sampling units (measuring 0.75 m × 2.5 m) arranged diagonally along the plot, with one sampling area allocated per crop row in a regularly spaced pattern (Figure 2). Only the last fully developed leafs of four potato plants were measured in each sampling unit. SPAD meter values were transformed into leaf chlorophyll content per leaf surface area (µg·cm^−2^) using the equation described by Uddling et al. [31], and values corresponding to one plot were averaged to give an estimate chlorophyll content at plot level. Authors suggesting equations to convert SPAD units to leaf chlorophyll content often arrive to different formulations, according to crop and environmental conditions, for example, and differences between converted values can be considerable even when equations used are derived for the same crop, as discussed by Parry et al. [32]. Despite that, considering the need in this study for data acquisition in the same plots over the season, non-destructive retrieval was preferred. Chlorophyll concentration at canopy level (g·m^−2^) was estimated per plot multiplying the average leaf chlorophyll content (derived from 16 values of chlorophyll content per plot, in each acquisition date) by the corresponding leaf area index (calculated based on 4 DHPs taken per plot, in each acquisition date, as described in Section 2.6), a method widely adopted to derive this attribute [33].

### 2.6. Leaf Area Index and Ground Cover

Leaf area index was estimated for each plot, after UAV data acquisitions, using an indirect non-destructive approach based on digital hemispherical photographs (DHPs) [34]. For this, DHPs were taken using a Nikon D-300s camera (12.3 MP; Nikon Corp., Tokyo, Japan) equipped with full-frame fisheye lens (Fisheye-Nikkor, focal length of 10.5 mm, maximal aperture f/2.8) looking downward and at approximately 0.6 m from the top of the canopy. Camera focus was set to infinity and ISO to 200, while aperture and shutter speed were adjusted automatically, to mitigate saturation issues due to overexposure [35]. Inside the plots, four pictures were acquired in each acquisition date following the same diagonal pattern described previously (Figure 2). The camera was manually levelled before shooting with aid of a bubble level attachment and images were registered in fine jpeg format. Camera optical centre and projection function, used to convert image pixels to zenith view angles, were estimated based on the method described by Baret [36]. Segmentation of each picture in vegetation and background components (i.e., potato canopy and soil) was performed through threshold optimization after transformation of image colors, as proposed by Liu et al. [37] and Song et al. [38].

Results of the picture segmentation procedure were divided in angular sectors with zenith and azimuth resolutions of 2.5 and 5°, respectively, in order to describe the different view angles registered in the images. Canopy gap fraction for each sector of the images was then derived and, due to characteristics of the lens used, only the angular interval between 0 and 37.5° was sampled. Leaf area index was estimated based on the relation described by Nilson [34,39], through the so called Poison model, between gap fraction, leaf area index and mean projected leaf area (G) in the plane perpendicular to the zenith view angle, considering uniform projection for different azimuth directions [35,40]. G is directly dependent on the leaf inclination distribution function (LIDF), described here using an ellipsoidal density function [41], which allows a flexible and simple representation of the LIDF requiring only one parameter (i.e., the ratio of vertical to horizontal projections of a representative volume of the canopy—χ). Through the relation between χ and the average leaf inclination of the canopy, the inversion of the Poison model to estimate leaf area index from canopy gap fraction becomes only dependent on the average leaf inclination angle, besides on the leaf area index itself.

In the method briefly described above, a random spatial distribution of leafs in the canopy is assumed, which differs from reality especially for row crop systems. A modified version of the Poison model was proposed by Nilson [39,42] to account for canopy non-random distribution through a clumping parameter (Ω), allowing to describe the relation of gap fraction with “true” values of leaf area index and average leaf inclination angle. The logarithm gap fraction averaging method, introduced by Lang and Xiang [43], was used to estimate the clumping parameter (Ω) for a given zenith view angle (θi) (Equation (1)):

(1) $$\Omega \left(\theta_{i}\right)=\frac{\ln\left\{\frac{1}{N}\sum_{j=1}^{N}\frac{1}{N_{\varphi }}\sum_{\varphi =1}^{N_{\varphi }}\left[P_{\varphi }\left(\theta_{i}\right)\right]\right\}}{\frac{1}{N}\sum_{j=1}^{N}\frac{1}{N_{\varphi }}\sum_{\varphi =1}^{N_{\varphi }}\ln\left[P_{\varphi }\left(\theta_{i}\right)\right]},$$

where N corresponds to the number of images taken in one plot and N_φ_ to the number of cells describing azimuth view angle variation for a given zenith angle. This approach relies on the fact that leaf area index is related to natural logarithm of the gap fraction and therefore the ratio between the logarithm of average gap fraction (large-scale component) and the average of gap fraction logarithm (small-scale component) gives a good estimate of canopy clumping [44,45]. In this approach, canopy elements are assumed to be randomly distributed within each individual cell.

The Poisson model inversion procedure allows to use a limited range of zenith view angles, a desirable feature considering that extreme view angles of DHPs can have higher degrees of targets mixing and masked areas, and, at the same time, the complete angular range is not always sampled, depending on the specifications of the camera-lens set. Several approaches were proposed to perform inversion of the Poisson model based on multangular gap fraction values [34]. In this study a method based on a look-up-table (LUT) was used [34,35,40]. For that, a LUT was built combining leaf area index values from 0 to 10 m^2^·m^−2^ (in 0.01 m^2^·m^−2^ steps) with average leaf inclination angles from 10° to 80° (with 2° intervals) and clump parameter estimates for all values of effective leaf inclination angle from 10° to 80°, with 2° intervals. In the case of the clumping factor, the effective leaf inclination angles were used to estimate saturated gap fractions (considering LAI at saturation level equal to 10 m^2^·m^−2^), which in turn were used to replace gap values for cells completely covered by vegetation. The gap averaging calculations were weighted by the amount of non-masked pixels in each cell. The cost function described by Demarez et al. [35] was used to retrieve the canopy parameters in the LUT that best described the gap fractions measured in the different zenith view angles in a plot.

Results of model inversion (i.e., estimates of leaf area index, average leaf inclination angle and clumping index) corresponded not only to leafs but to all vegetation components, since photosynthetically active elements could not be distinguished from other plant parts like senescent leafs, branches and stems [40]. Therefore, the so called plant area index is the parameter derived through this inversion and in subsequent sections the term leaf area index refers to plant area index adjusted for clumping effects.

Ground cover at plot level was estimated based on the ratio between foreground and background components derived from the segmentation of DHPs, considering only the zenith view angle interval between 0° and 20°.

### 2.7. Vegetation Indices

Vegetation indices were calculated from UAV and ground-based spectra, at plot and intra-plot levels, for all acquisition dates. From the numerous vegetation indices designed to describe different properties of leaf and canopy, those listed in Table 2 were selected to explore the relationship between crop traits and spectral information during the growing season. The normalized difference vegetation index (NDVI) is widely used due to its relation with amount of vegetation and photosynthesis capacity, light use efficiency and biomass productivity, while reducing the effects of illumination conditions on spectral data [46]. However, it is largely affected by soil reflectance (background effects) and has low sensitivity to changes of plant properties when the sensed area is completely covered by vegetation. Indices searching to correct for background effects as the weighted difference vegetation index (WDVI) often take advantage of the constant relationship between soil reflectance in red and near-infrared wavelengths, if soil moisture is the only changing factor [47,48]. WDVI is highly correlated with canopy structural properties like leaf area and can be described as an orthogonal index since it is given as a function of isolines parallel to the principal axis of the soil spectral variation in the red versus near-infrared space (soil-line) [49]. Other vegetation indices were developed considering the soil-line concept to adjust for background effects in estimations of vegetation properties, like the optimized soil-adjusted vegetation index (OSAVI) [46].

Various indices have been designed specifically to estimate leaf chlorophyll content as the modified chlorophyll absorption in reflectance index (MCARI), which searches to reduce effects of background and non-photosynthetic materials in the spectral measurements [20]. MCARI was developed from CARI, proposed by Kim et al. [50], and was further improved by Haboudane et al. [51] in order to reduce residual effects of non-photosynthetic elements at low chlorophyll concentrations, originating the transformed chlorophyll absorption ratio index (TCARI). Also, following the integration of MCARI with OSAVI proposed by Daughtry et al. [20], the same combination can be applied to TCARI to reduce influence of soil reflectance resulting in two different indices, MCARI/OSAVI and TCARI/OSAVI [52]. Wu et al. [52] proposed to replace bands in these indices at 670 nm and at 700 or 800 nm by bands centered at 705 and 750 nm, respectively. They verified that linearity of the relationship with leaf chlorophyll content improved while sensitivity to changes in canopy structural properties decreased after bands in the red edge region were integrated. Ciganda et al. [53] and Clevers and Kooistra [4] also demonstrated that an index based on a spectral band in the red edge region, the red edge chlorophyll index (CI_re_) [54], has linear relation to leaf chlorophyll and nitrogen contents being well suited for estimations of these properties.

The inflexion point of vegetation spectra in the red edge region (between 680 and 780 nm), referred as red edge position (REP), has proven to be a measure sensitive to vegetation properties at leaf and canopy levels like chlorophyll content and leaf area. However saturation of this index under high chlorophyll content scenarios has been reported [48]. The REP was estimated in this study using the linear four-point interpolation method [55,56]. This approach consists of estimating the reflectance in the red edge point as the average of the reflectance in the near-infrared (780 nm) and in in the region of maximal energy absorption by chlorophyll (680 nm). The final REP value is calculated proportionally in relation to reflectance in wavelengths between 700 and 740 nm.

Based on the reflectance spectra slope in the red edge region, the MERIS terrestrial chlorophyll index (MTCI) was developed as a relatively simple approach to estimate changes in the red edge position using bands included in the Medium Resolution Imaging Spectrometer (MERIS) sensor [57]. Although designed to be derived from discontinuous spectral information it has been described as having better performance than REP or other red-edge-based indices for chlorophyll estimation [58], especially for high chlorophyll contents, even when compared with indices designed to be derived from continuous narrow spectral bands.

Gitelson et al. [54,59] found that replacing a band in the red edge region (710 nm) by a band in the green region (550 nm) in an index with same format as CI_re_ would bring comparable result in anthocyanin-free leaves and proposed the green chlorophyll index (CI_g_) as alternative to estimate leaf chlorophyll. Besides chlorophylls, other leaf pigments are related to photosynthetic activity and participate in the regulation of radiation use by plants. The photochemical reflectance index (PRI), also using spectral bands in the green region, has been proposed as indicative of the epoxidation state of xanthophyll cycle pigments, which are related to the efficiency of photosynthesis [60]. Although it has a strong relationship at leaf level with xanthophylls, for longer time periods PRI is sensitive to other leaf pigments, like carotenoids and to the ratio of carotenoids versus chlorophylls concentration [61,62,63,64], besides being sensitive to changes in canopy structure [65,66], like variations in leaf area, facts that need to be taken into account for vegetation monitoring over time.

Finally, to improve prediction of leaf area Haboudane et al. [67] introduced the MCARI2, in which a band in the red region (700 nm) present in the original MCARI was replaced by a band in the near-infrared (800 nm) and a soil adjustment factor was added, to reduce chlorophyll, background and atmosphere effects while increasing linearity of the relationship with leaf area.

### 2.8. Crop Traits Modelling and Intercomparison of Different Spectral Acquisition Levels

Vegetation indices derived from UAV and ground-based measurements were used as predictors to model crop properties, using linear regressions fitted through ordinary least squares. The predictive performance of each model was assessed based on root mean squared errors (RMSE) and coefficient of determination (R^2^). RMSE was estimated using repeated (100 times) 10-fold cross-validation (with final RMSE values corresponding to the average for the 100 repetitions) while R^2^ was calculated for models fitted to the complete dataset. Confidence and prediction intervals (95% confidence level) were derived considering the complete dataset as well. Also, the mean errors (the sum of residuals divided by the number of observations) were evaluated per acquisition date (from 43 to 99 DAP). Estimations derived from the fitted models were used for their calculation.

The direct intercomparison of vegetation indices from both sensing approaches was performed fitting linear regressions to the values of each index and calculating R^2^ and discrepancy of slope (DS), which consists of subtracting from one the slope of the linear model considered [69]. R^2^ and DS were used as parameters to assess comparability of the outputs at plot level.

Finally, to assess UAV and ground-based sensors capabilities to sample comparable information and its discriminative potential concerning differences between treatments, the Bhattacharyya coefficient (B-coefficient; [70]) was used. For that, average and standard deviation were calculated in order to describe a normal distribution for values of each vegetation index corresponding to a given treatment. The B-coefficient was then derived as indicated in Equation (2):

(2) $$B-\mathrm{coeff}.\left(t_{1}{,t}_{2}\right)=\int \sqrt{t_{1}\left(x\right)t_{2}\left(x\right)}\mathrm{dx},$$

where t_1_ and t_2_ are the normal probability distributions of values corresponding to the different treatments (i.e., non-mixed—t_1_—and mixed varieties—t_2_), for a given vegetation index. The result was subtracted from one to give a parameter varying between zero and one, corresponding to totally overlapping to completely separated probability distributions, respectively.

## 3. Results

### 3.1. Description of Crop Development and Canopy Reflectance over Time

**Figure 3:**
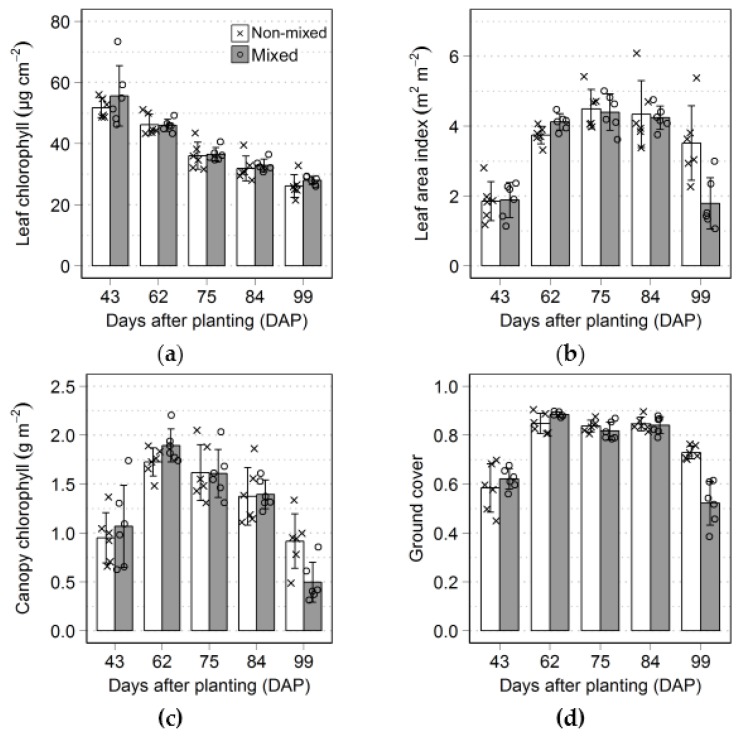
Vegetation properties at plot level for both production systems (i.e., non-mixed and with mixed varieties) over time (from 43 to 99 DAP): (**a**) Leaf chlorophyll content; (**b**) Leaf area index; (**c**) Canopy chlorophyll content; and (**d**) Ground cover. Dots represent observations for each treatment and error bars the standard deviation.

During the growing season crop traits were measured on different dates to describe leaf and canopy development (Figure 3). Leaf chlorophyll content decreased over time (Figure 3a), especially between the first (43 DAP) and the third (75 DAP) data acquisitions, while canopy structure was still developing, as indicated by changes of leaf area index and ground cover levels on this period (Figure 3b,d). This continuous decrease in leaf chlorophyll content is also observed for leaf nitrogen in potato and it is associated to biomass accumulation in shoots and tubers during the crop growth [71]. Ground cover arrived to its maximum after the second (62 DAP) data acquisition (Figure 3d) and only consistently decreased after the fourth (84 DAP) acquisition, towards the end of the growing season. Canopy chlorophyll content (Figure 3c) was apparently limited by lower canopy structure on the initial acquisitions (43 and 62 DAP) and by the decrease in leaf chlorophyll content during the third and fourth acquisitions (from 75 to 84 DAP), since changes in canopy structure were minimal during this period.

For the last dataset collected (99 DAP), no obvious differences were observed concerning leaf chlorophyll content between different production systems (Figure 3), although values describing canopy properties were consistently smaller for plots with mixed varieties. Between the fourth and the last data collections (i.e., 84 and 99 DAP), late blight was detected in all plots with mixed varieties, while those cultivated according to the non-mixed treatment were relatively less affected by the disease. The characteristics of the different varieties included in each treatment, concerning resistance to the pathogen, as described in Section 2.1, are most likely the causes of the differences between production systems. Although, apparently, leaf chlorophyll content was not affected by late blight incidence (Figure 3), only the last developed leafs were measured during the determination of this trait, while basal leafs in the canopy are known to be generally attacked earlier and more severely [72].

**Figure 4:**
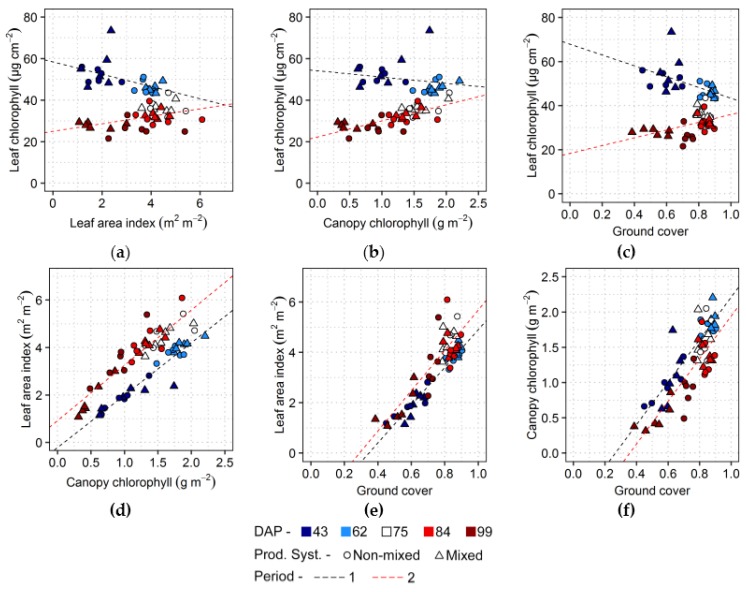
Pairwise comparison of crop traits measured over the season (from 43 to 99 DAP): (**a**) Leaf chlorophyll content and leaf area index, (**b**) Leaf chlorophyll content and canopy chlorophyll, (**c**) Leaf chlorophyll content and ground cover, (**d**) Leaf area index and canopy chlorophyll, (**e**) Leaf area index and ground cover, (**f**) Canopy chlorophyll content and ground cover. Linear trends (estimated by least squares) describing the relationship between properties are indicated for two periods: period 1, corresponding to the first two acquisitions (from 43 to 62 DAP); and period 2, comprising the last three acquisitions (from 75 to 99 DAP).

Comparison between traits observed during the growing season is presented in Figure 4. A negative trend occurred between leaf chlorophyll and leaf area index (Figure 4a) for observations corresponding to the first two acquisition dates (from 43 to 62 DAP). After that, leaf area index changed very little, except on the last acquisition when considerable lower leaf area levels were observed in comparison with the previous dates, in particular for plots with mixed varieties, while leaf chlorophyll content continued to decrease, resulting in a positive trend between these traits in this period, as can be observed in Figure 3 as well. Conversely, leaf chlorophyll had a relatively weaker relationship with canopy chlorophyll during the first two acquisitions (from 43 to 62 DAP; Figure 4b) and a stronger positive relation after this period (from 75 to 99 DAP). Canopy chlorophyll content was always positively associated to leaf area index (Figure 4d), although in the period between the third and the last data collections (from 75 to 99 DAP) a given content of canopy chlorophyll corresponded to higher levels of leaf area index in comparison with the previous period, indicating that the canopy structure was more developed during period 2. A similar trend was described by Gitelson et al. [73] for the same traits (i.e., canopy chlorophyll and leaf area) in maize, despite physiological differences between maize and potato. They observed a linear relation during the vegetative stage and a slightly nonlinear trend during reproductive stage, matching the periods 1 and 2 described in Figure 4d, since potato flowering started approximately in the third acquisition (Table 1). Ground cover was positively related to other canopy properties (i.e., leaf area index and canopy chlorophyll; Figure 4e,f) in both periods considered. Association of ground cover with leaf chlorophyll (Figure 4c) was similar to that observed between leaf area index and leaf chlorophyll, but after the two initial observations ground cover achieved its maximum and observations were less disperse, especially for the third and fourth acquisitions (75 and 84 DAP).

**Figure 5:**
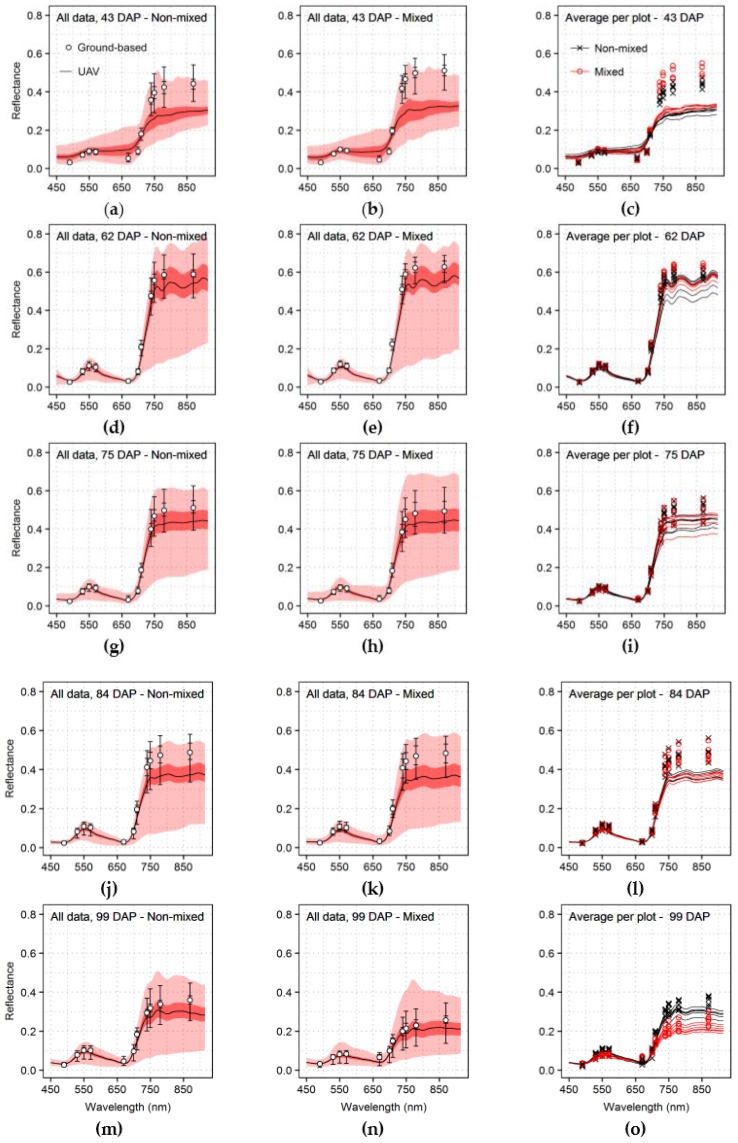
Description of the spectra acquired using ground-based (dots) and UAV sensor (lines) for each production system (i.e., non-mixed and with mixed varieties) and acquisition date: (**a**,**b**) 43, (**d**,**e**) 62, (**g**,**h**) 75, (**j**,**k**) 84 and (**m**,**n**) 99 DAP. Data range (maximum and minimum values), 25% and 75% quartiles are represented using red shades for the complete UAV dataset and using error bars for all ground measurements. Graphs in last column (**c**,**f**,**i**,**l**,**o**) correspond to the averaged reflectance for plots of both cultivation methods.

The spectral measurements acquired during the crop growth using both sensor systems are summarized in Figure 5. Reflectance was generally lower in wavelengths affected by absorption from leaf pigments, between 450 and 700 nm, during the first acquisition date (43 DAP) for ground-based measurements, in comparison with UAV spectral information. During the same acquisition, reflectance for bands above 700 nm (near-infrared), which are related to light scattering by leaf and canopy structural components [48,62,67], was higher for ground-based readings as well. This indicates that ground-based sensing allowed to capture a stronger vegetation signal, in these first measurements, most likely because ground-based reflectance was measured directly over the crop rows reducing the fraction of canopy gaps in the sampled area.

Although on subsequent measurements, until 84 DAP (fourth data acquisition), the same trend of stronger vegetation signal for ground-based measurements was still present, UAV and ground data were more comparable, particularly for the second and third acquisitions (62 and 75 DAP), with average ground-based reflectance within the upper quartile of UAV spectral data (Figure 5d,e,g,h). Reflectance corresponding to ground-based and UAV sensor started to differ more intensely during the fourth acquisition (84 DAP), especially for wavelengths above 700 nm, indicating a possible reduction in canopy structure (Figure 5l), with greater impact on UAV-based data. 

On the last acquisition (99 DAP), reflectance in wavelengths from 450 to 700 nm, a region in which incident energy is mostly absorbed by leaf pigments, increased while it decreased in the near-infrared, region related to scattering by canopy structure, for both sensors, especially for the mixed production system (Figure 5m–o). These are effects of canopy structure loss coupled with reduction in the chlorophyll content, as indicated by data described in Figure 3, and can be attributed to both natural vegetation senescence and late blight incidence, especially upon plots with mixed varieties. Furthermore, plots cultivated with the non-mixed system had slightly lower vegetation signal on the first (43 DAP) and second (62 DAP) acquisitions (Figure 5c,f) due to lower leaf chlorophyll content and smaller canopy structure (Figure 3), dissimilarities most likely related to intrinsic characteristics of each potato variety included in the different production systems. Additionally, the strongest vegetation signal, in comparison with other dates, was observed during the second acquisition (62 DAP; Figure 5d–f), which can be attributed to the relatively higher canopy chlorophyll levels (Figure 3) coupled with specific illumination and sun-target-sensor geometry in this specific date. The spectral readings corresponding to the second acquisition were measured under overcast conditions and with low sun elevation (Table 1), in comparison with other dates, factors that can have reduced contributions from background and shadowing effects to the reflectance at canopy level, increasing the characteristic vegetation signal [21,74,75].

### 3.2. Performance of Crop Traits Retrieval Using UAV and Ground-Based Spectral Measurements

Results from linear regressions relating vegetation indices to crop properties are presented in Table 3. Considering that data from all acquisitions were used together to build the predictive models, results indicate that relatively accurate estimations could be obtained for crop properties, despite possible changes in background reflectance, sun-target-sensor geometry and illumination conditions between acquisitions. UAV data yielded the best predictions in comparison with ground measurements, regardless the potentially higher mixing of soil and vegetation signals at plot level. The best estimates for leaf chlorophyll content using UAV data were achieved using TCARI/OSAVI, which compensate for effects of background reflectance (as indicated in Section 2.7). For ground-based measurements, MTCI, an index sensitive to chemical and structural properties at leaf and canopy levels, performed comparatively better.

**Figure 6:**
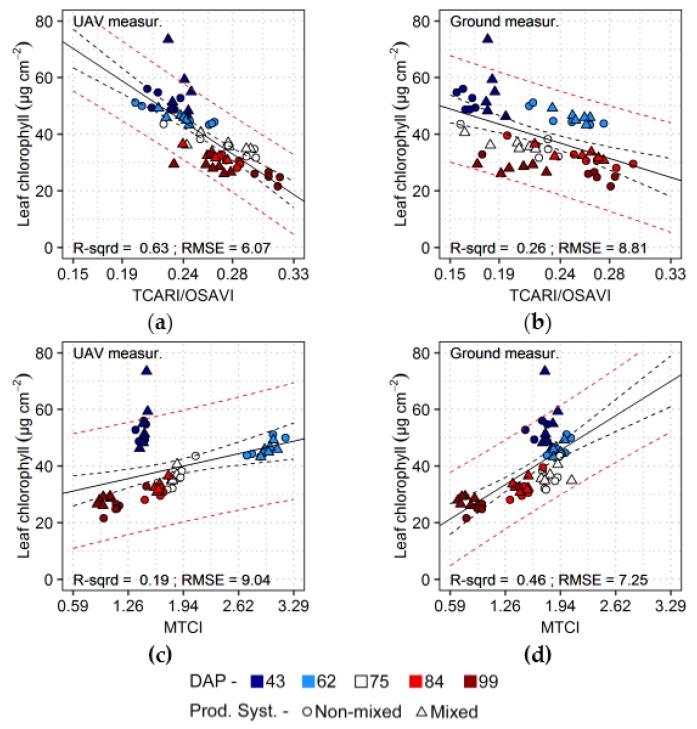
Best vegetation indices for prediction of leaf chlorophyll content from UAV and ground-based data (**a**,**d**, respectively) and corresponding results for ground-based and UAV measurements (**b**,**c**, respectively). Red and black dashed lines indicate prediction and confidence intervals with 95% confidence level, respectively.

In Figure 6, scatter plots corresponding to the vegetation indices yielding the lowest RMSE for predictions of leaf chlorophyll content are presented. The most evident differences between relationships derived for UAV and ground-based data concern observations from the first (43 DAP) and second (62 DAP) acquisitions. Spectral measurements corresponding to 43 DAP were affected by background reflectance, with apparent greater impact upon data from the UAV sensor as can be observed for values of MTCI (Figure 6c,d). In opposition, TCARI/OSAVI seems to have mitigated background effects yielding comparable outputs between both sensing levels for the first acquisition (Figure 6a,b). Observations from 62 DAP deviate from others when TCARI/OSAVI values corresponding to ground-based measurements are considered (Figure 6b). According to Wu et al. [52], TCARI/OSAVI is especially sensitive to changes in leaf area index during initial canopy development (i.e., leaf area index values lower than 3 m^2^·m^−2^). This may have caused the shift from lower to higher values for this index when comparing ground-based data from the first (43 DAP) and second (62 DAP) acquisitions, respectively, considering that leaf chlorophyll content changed considerably less than leaf area index during this period (Figure 3). On the other hand, comparing values of TCARI/OSAVI from ground-based and UAV measurements for the second acquisition (62 DAP) (Figure 6a,b), lower values occurred for UAV outputs, a difference probably related to illumination conditions and contributions from soil reflectance. As observed in Section 3.1 as well, the second acquisition happened under overcast conditions (i.e., increased diffuse incoming radiation) and larger sun azimuth angle than in other dates, which may have affected canopy reflectance. These factors can potentially change the fraction of shaded versus sunlit vegetation and the interaction of the incoming energy with canopy and background elements [21,74]. Therefore, together with contributions from soil reflectance, it may have induced a shift of TCARI/OSAVI towards lower values for UAV observations corresponding to 62 DAP. The same illumination conditions apparently had a lower impact on ground-based measurements, which seem to be mainly related to changes in leaf chlorophyll and leaf area during this specific acquisition.

Observations corresponding to the second acquisition (62 DAP) resulted also in comparatively higher values of MTCI (Figure 6c), specially for UAV measurements, which may be explained by the same illumination aspects described before, besides the high levels of canopy chlorophyll content. Apparently, illumination had lower influence upon ground-based measurements in this case, as observed also for values of TCARI/OSAVI.

**Figure 7:**
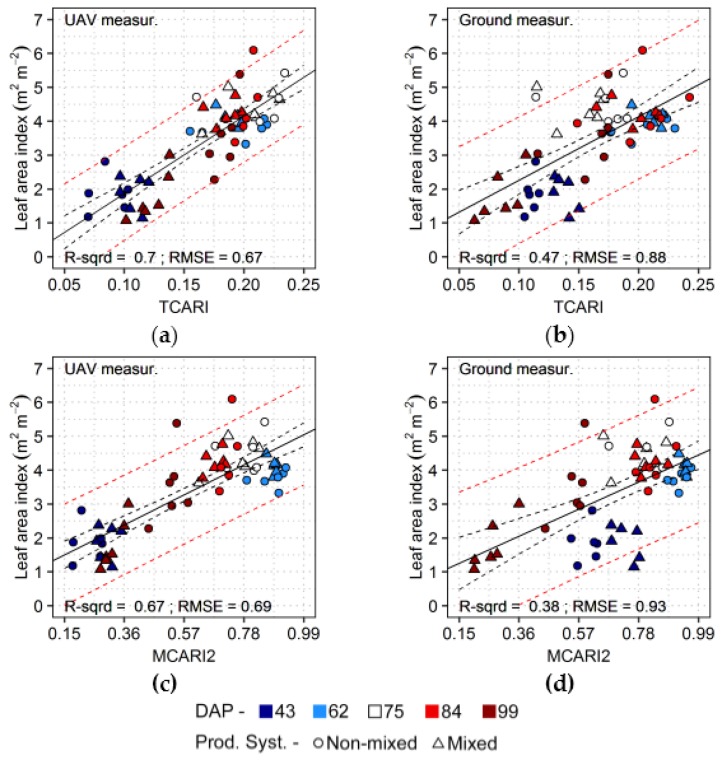
Best vegetation index for prediction of leaf area index from UAV data (**a**), results for an index designed to predict leaf area index (**c**), and corresponding results for ground-based measurements (**b**,**d**). Red and black dashed lines indicate prediction and confidence intervals with 95% confidence level, respectively.

Estimates of leaf area index through UAV data were more accurate using TCARI (Table 3), which is an index proposed to predict leaf chlorophyll content [51], despite still being sensitivity to changes in canopy structure and to soil reflectance [51,52,76,77]. For ground measurements, MCARI performed better than other indices, but results were similar to those corresponding to TCARI. In the scatter plot in the Figure 7, a comparison of TCARI with MCARI2, an index optimized to predict leaf area index, indicates that the former was able to better group together, for both sensor systems, measurements from the second (62 DAP) to the fourth (84 DAP) acquisitions. During this period comparable leaf area index levels were observed and grouping observations corresponding to these dates increased accuracy. Besides that, TCARI was apparently more sensitivity to background reflectance for the first acquisition in comparison to MCARI2, as can be observed for ground-based measurements (Figure 7b,d), increasing prediction accuracy in this case. However, MCARI and TCARI saturate fast for increases in leaf area, and this factor could hinder accurate estimates in datasets with larger dynamic ranges for this attribute than that observed here [52]. 

**Figure 8:**
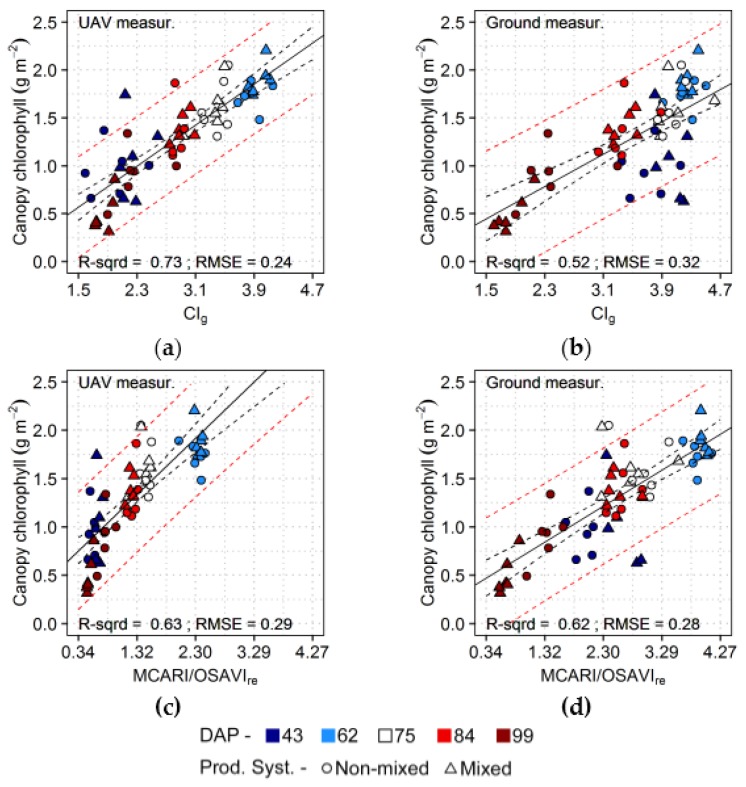
Best vegetation indices for prediction of canopy chlorophyll content from UAV and ground-based data (**a**,**d**, respectively) and corresponding results for ground-based and UAV measurements (**b**,**c**, respectively). Red and black dashed lines indicate prediction and confidence intervals with 95% confidence level, respectively.

Chlorophyll content at canopy level was relatively well predicted based on values of CI_g_ derived from UAV data (Table 3), with homogeneous residuals for different acquisition dates (Figure 8). Similarly, ground-based measurements resulted in relatively accurate estimations of canopy chlorophyll, but in this case lower retrieval errors were achieved using MCARI/OSAVI_re_. CI_g_ calculated using ground-based spectra overestimated canopy chlorophyll for observations from the first acquisition (43 DAP). This index was better related to leaf chlorophyll in this case (Table 3). Gitelson et al. [78] and Viña et al. [79] described a linear relationship between CI_g_ and canopy properties (i.e., leaf area and canopy chlorophyll content) for measurements corresponding to relatively larger areas (i.e., around 2.4 m radius of sensor footprint) in comparison with the ground-based data used here. Conversely, characteristics of the dataset used by these authors match the UAV measurements collected in this study, after integration at plot level. In this respect, results obtained by these authors corroborate those presented here indicating the potential of CI_g_ for predictions of chlorophyll content at canopy level, even under influence of considerable amount of background reflectance.

**Figure 9:**
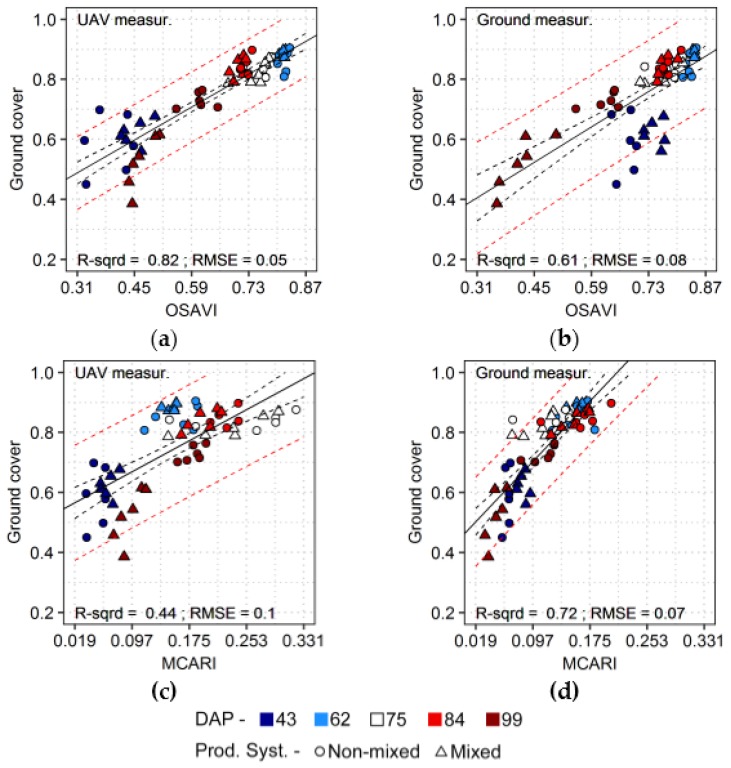
Best vegetation indices for prediction of ground cover from UAV and ground-based data (**a**,**d**, respectively) and corresponding results for ground-based and UAV measurements (**b**,**c**, respectively). Red and black dashed lines indicate prediction and confidence intervals with 95% confidence level, respectively.

For ground cover estimation, OSAVI was the best option when information derived from UAV-based sensor was used (Table 3, Figure 9). OSAVI is an index associate to leaf and canopy properties with compensation for background effects (as described in Section 2.7). Indices optimized to predict leaf area index, like MCARI2 and WDVI, or with general sensitivity to leaf and canopy traits, like NDVI, also resulted in relatively accurate predictions of ground cover using this dataset. OSAVI overestimated fractional cover for ground-based measurements corresponding to the first acquisition (43 DAP, Figure 9b). This overestimation was regularly observed for ground-based spectra measured during the first acquisition, when properties at canopy level were predicted. It may indicate that the preferential sampling adopted for the ground-based measurements yielded a worse relation with canopy properties, at least when compared with integrated ground truth data, as observed for leaf area index and canopy chlorophyll estimates as well (Figure 7 and Figure 8, respectively). MCARI performed better for prediction of fractional cover from ground-based data, as observed for leaf area index as well. As discussed before, this index is sensitivity to bare soil [51,52] and this may have helped to increase quantification potential in this case, when models were fitted to observations from all acquisitions dates.

**Figure 10:**
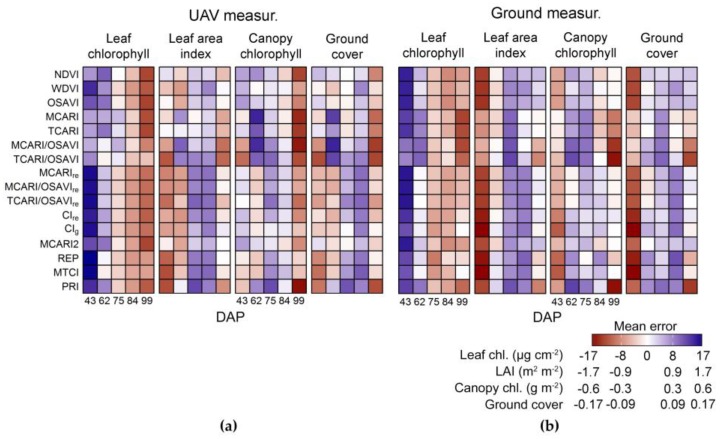
Mean error of linear models relating vegetation indices to crop traits for each acquisition date (from 43 to 99 DAP). Vegetation indices calculated from UAV (**a**) and ground-based measurements (**b**).

Mean prediction errors associated to each sensing approach and acquisition date (from 43 to 99 DAP) are described in Figure 10. For leaf chlorophyll content, observations corresponding to the first data collection were in general underestimated by the predictive models (i.e., positive mean errors), especially when vegetation indices based on wavelengths in the red edge region were used with UAV data (Figure 10a). The same occurred for ground-based spectra but indices based on bands in the red edge performed better for this dataset (Table 3, Figure 10b). Estimations of leaf chlorophyll for observations corresponding to the last data collection were in general overestimated (i.e., negative mean errors) by models fitted to UAV data, especially by those derived from indices without bands in the red edge region (Figure 10a). The same overestimation corresponding to the last data acquisition was observed for ground-based measurements but mainly when MCARI, TCARI and combinations of these with OSAVI were used (Figure 10b).

For leaf area index, observations corresponding to the first (43 DAP) and second (62 DAP) acquisitions were in general overestimated while those from the third (75 DAP) and fourth (84 DAP) data collections were underestimated, when UAV data was used (Figure 10a). In this case, uniform errors were observed for indices with broad sensitivity to leaf and canopy properties, i.e., NDVI and OSAVI, or with specific sensitivity to leaf area, i.e., WDVI and MCARI2. Additionally, MCARI and TCARI provided predictions based on UAV measurements with the lowest mean errors for different dates, which were also relatively uniform, despite not being designed to predict leaf area index. For ground-based data, leaf area index corresponding to the first acquisition (43 DAP) was generally overestimated, but at lesser extent by models based on MCARI, TCARI or combinations of them with OSAVI (Figure 10b). Also for predictions derived from ground-based measurements, leaf area index was underestimated towards the end of the season, between 75 and 84 DAP (Figure 10b), as observed for UAV data as well.

Quantification of canopy chlorophyll through both sensing approaches generally resulted in uniform residuals for predictions corresponding to different acquisition dates (Figure 10b), especially for indices including bands in the red edge region. MCARI, TCARI and combinations of those with OSAVI yielded considerable worse estimations, when red edge bands were not included in their formulation, with underestimation for predictions corresponding to the second (62 DAP) and third (75 DAP) acquisitions and overestimation for the last data collection (99 DAP), considering both datasets.

Predictions of ground cover from UAV data were more accurate using indices sensitive to vegetation properties at leaf and canopy levels, i.e., NDVI and OSAVI, or indices designed to estimate leaf area index, i.e., WDVI and MCARI2 (Figure 10a). Similarly, fractional cover was well predicted using NDVI, OSAVI, WDVI or MCARI2 with ground-based data, but in this case MCARI and TCARI provided the best performances, with more uniform residuals between different acquisition dates (Figure 10b).

Considering the general distribution of the prediction errors, indicated in Figure 10, observations corresponding to the first acquisition (43 DAP) are those with consistently higher residuals, in comparison with other dates, in particular for estimates of leaf chlorophyll content. This acquisition date was characterized by lower canopy development and higher leaf chlorophyll content (Figure 3), originating a set of discrepant observations with more intense contribution from background reflectance especially for UAV-based measurements. Fitting prediction models to data from all acquisitions except those observations corresponding to 43 DAP resulted in lower residuals for estimates of leaf chlorophyll, canopy chlorophyll and ground cover (Table 4; Table A1), for both datasets. Only for predictions of leaf area index based on UAV spectra RMSE was smaller using the complete set of observations (Table 3 and Table 4), although, in this case, RMSE was almost the same of that achieved using a reduced dataset, and corresponding ground-based estimations of leaf area index were considerably more accurate without observations from the first acquisition date.

### 3.3. Intercomparison of UAV and Ground-Based Spectra

**Figure 11:**
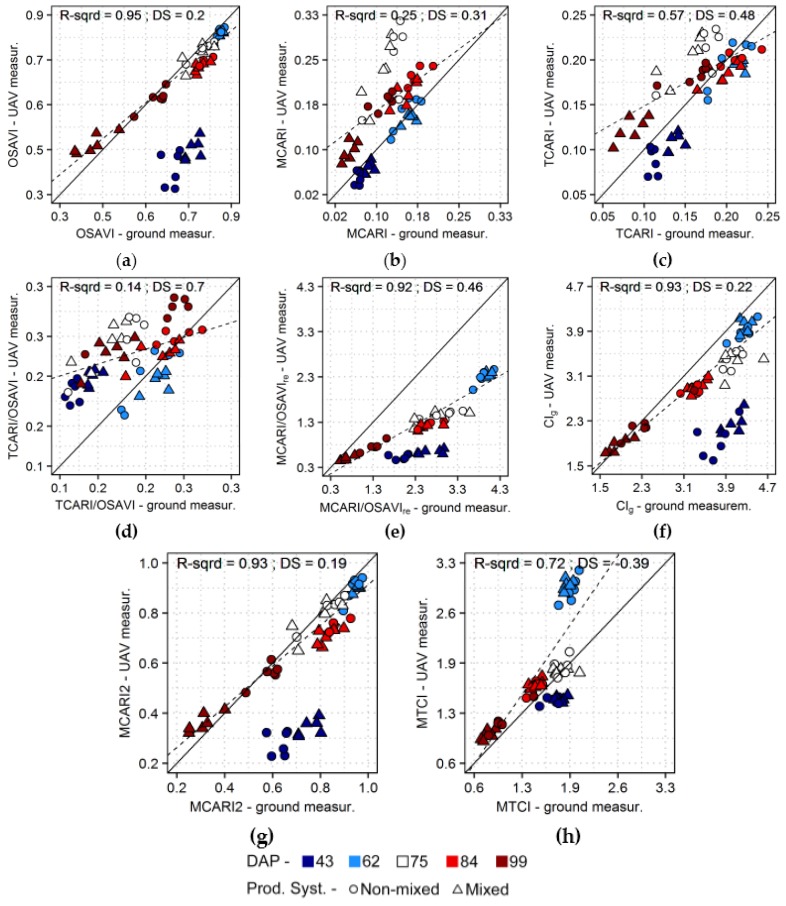
Intercomparison of spectra acquired with handheld and UAV-based spectrometers, for the vegetation indices with the best performace: (**a**) OSAVI, (**b**) MCARI, (**c**) TCARI, (**d**) TCARI/OSAVI, (**e**) MCARI/OSAVI_re_, (**f**) CI_g_, (**g**) MCARI2 and **(h**) MTCI. R^2^ and DS parameters, as well regression lines, corresponding to linear models fitted only to the last 4 acquisitions (from 62 to 99 DAP). DS indicates proximity of the linear relation between sensors to the 1:1 line, with zero meaning that this relation corresponds exactly to the 1:1 line.

Comparison of vegetation indices calculated using data acquired at different levels are presented in Figure 11, for indices yielding the best estimates concerning the different crop traits evaluated in this study (Table 3 and Table 4). Indices based on contrasts between absorbance in the visible wavelengths (up to 700 nm) and scattering in the near-infrared region, like OSAVI, CI_g_ and MCARI2 (Figure 11a,f,g), resulted in comparable outputs between datasets, after canopy maximal structural development was achieved (i.e., after 62 DAP). Conversely, indices based only on wavelengths in the visible region, like MCARI and TCARI, and their combination with OSAVI (Figure 11b–d) yielded considerably divergent values between different sensing approaches. Indices exploring the red edge region, like MCARI/OSAVI_re_ and MTCI (Figure 11e,h), resulted in comparable outputs between sensors but these indices were apparently more affected by illumination aspects, specially MTCI, as indicated by the deviation from linearity for observations corresponding to the second data collection (62 DAP), which was performed under overcast conditions and low sun elevation. A constant bias towards ground-based measurements occurred for CI_g_ and, particularly, for MCARI/OSAVI_re_, most likely because ground-based readings were less affected by background reflectance since they were made with the sensor pointing to the center of the crop rows. Values corresponding to the first acquisition (43 DAP) had a general offset in comparison with those observed on other dates, for all indices, but especially for formulations exploring spectral changes in the visible in contrast to near-infrared region, like OSAVI, CI_g_ and MCARI2, and for indices based on bands in the red edge region, like MCARI/OSAVI_re_ and MTCI. Observations from the first acquisition also had an offset when considering MCARI and TCARI (i.e., higher values for ground-based data), but in this case residuals were relatively high for all acquisitions, probably due to an interaction between changes in environmental conditions (i.e., illumination and sun-target-sensor geometry) and vegetation properties (i.e., leaf and canopy traits) with the characteristics of each sensor (e.g., spatial support, information sampling, etc.).

**Figure 12:**
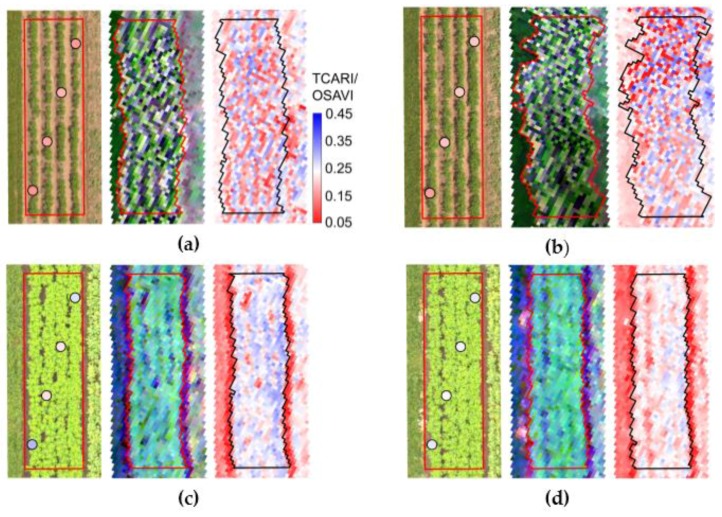

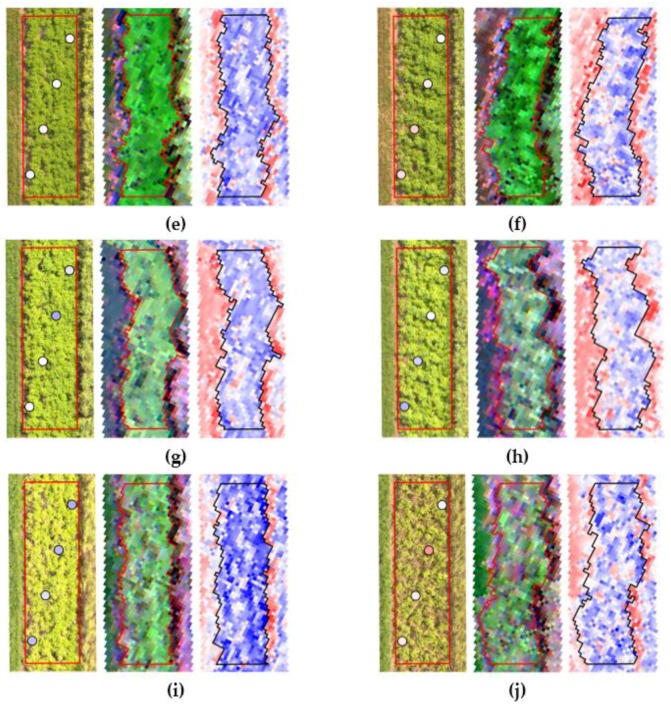
RGB orthomosaics (derived from images taken with the photogrammetric camera, as described in Section 2.2), true color composite from hyperspectral images (bands centered at 665, 555 and 485 nm as RGB layers, respectively) and values of TCARI/OSAVI for one plot from non-mixed (**a**,**c**,**e**,**g**,**i**) and mixed production systems (**b**,**d**,**f**,**h**,**j**), from 43 (**a**,**b**) to 99 DAP (**i**,**j**). Points over the orthoimages represent TCARI/OSAVI calculated from ground-based measurements and colors follow the same scale of pixel-wise results corresponding to the hyperspectral images.

In the Figure 12, images corresponding to one plot cultivated with the non-mixed production system and another with mixed varieties, both located in the second block of the experiment, are presented for all acquisition dates (from 43 to 99 DAP). In general, concerning the crop development over the season, it can be observed that both production systems resulted in similar vegetation growth from 43 (Figure 12a,b) to 84 DAP (Figure 12g,h), and dissimilarities started to be perceptible only on the last acquisition, resulting mainly from more intense late blight spread in plots under mixed production system, as indicated by crop traits measured on this date (Figure 3), i.e., lower levels of canopy structural traits for plots of the mixed system.

Also, in the images acquired with the hyperspectral sensor (Figure 12) variable internal geometry quality was observed over the season, as described in Section 2.3. In some cases, this fact probably reduced accuracy of the spectral information retrieved for a given area, and it may be of especial importance for studies or mapping efforts concerning characterization of small plots, as in phenotyping studies. Difficulties in geometric correction procedures involving data acquired by pushbroom sensors on board of UAV systems are described by other authors [80,81] and must be considered when choosing the most suitable imaging system and pre-processing procedures for a given context. In Figure 12, the variation in the geometric distortions observed over time are probably related to changes in wind speedy and/or direction during each data acquisition. Therefore, besides being a limiting factor for the flight realization, wind condition can impact quality and spatial accuracy of the information acquired [82].

Over the RGB orthoimages in Figure 12, values of TCARI/OSAVI derived from the point-based ground measurements are presented and can be compared with the pixel-wise values of the same index calculated for the corresponding hyperspectral images. From this comparison, it is possible to observe that considerable part of the intra-plot variability was apparently not sampled using the point-based sensor. Although averaged information at plot level from middle to late season resulted in comparable performance for crop traits quantification, as indicated in Section 3.2, and in similar values and trends for some of the vegetation indices evaluated in this study (Figure 11), if intra-plot or fine scale variability are of interested in a given context different sensing approaches might lead to different conclusions. This aspect can be of special importance when information acquired using a “selective configuration”, i.e., with sensor pointing to a specific location over the mapped area is compared with an exhaustive “not preferential” data source.

**Figure 13:**
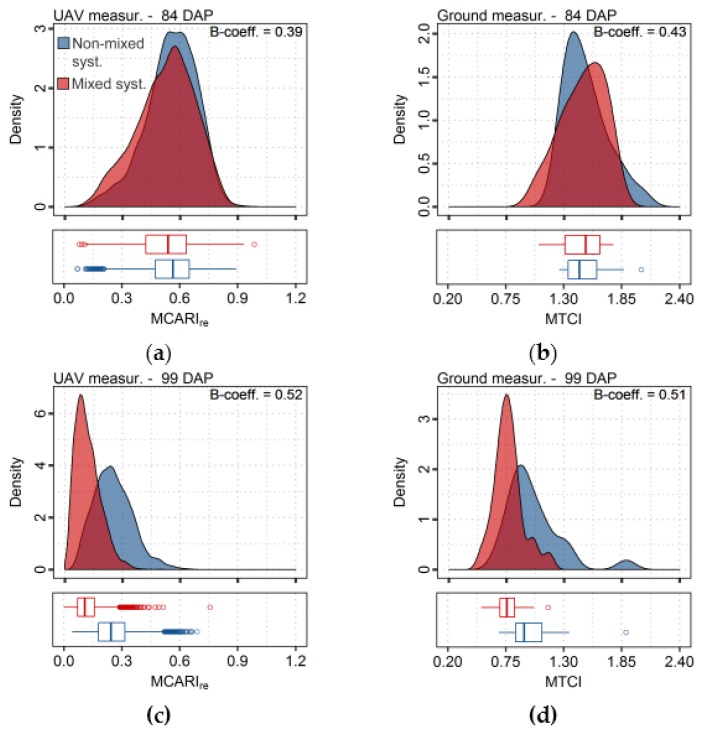
Distribution of MCARI_re_ and MTCI values calculated from UAV (**a**,**c**) and ground-based data (**b**,**d**), respectively. Estimate probability density and boxplot corresponding to observations from a given sensor and acquisition date are presented for each treatment (i.e., non-mixed and mixed systems). Results concerning UAV observations correspond to approximately 10,800 pixels and those derived from ground data represent 48 spectral measurements, per acquisition date.

For this reason, in order to assess if data from both sensing approaches evaluated here yielded similar information sampling and discrimination potential for differences observed between production systems, a comparison of vegetation indices distributions is presented in Figure 13 and values of a parameter (B-coefficient) corresponding to this comparison is provided in Table A2. These results show that indices with the best discrimination potential for each sensor achieved a comparable potential for segregation of treatments, especially for the last data acquisition (99 DAP), in which treatments were the most dissimilar in comparison with other dates due to stronger late blight incidence upon plots with mixed varieties. Distributions of vegetation indices for an acquisition date before the disease outbreak (84 DAP) indicate that no obvious differences between treatments is detected by any sensing method in this case, following the trend observed for crop traits (Figure 3), which showed comparable levels of crop development between both production systems.

The vegetation indices providing the best discriminative potential between treatments for UAV and ground-based measurements, MCARI_re_ and MTCI, yielded good estimates of leaf and canopy properties, especially for chlorophyll content (Table 3, Table A1). This indicates that indices describing not only leaf properties but also canopy traits resulted in better segregation of crop with this specific late blight incidence level, in relation to relatively healthier plants. Plots with mixed varieties presented disease severity varying between 25% and 75% of leaf area dead per plot, on the last acquisition date. At this infection stage, not only leaf biochemical composition was affected, by the pathogen development, but also structural properties at canopy level, and therefore indices describing global canopy health status were more effective for treatments segregation.

## 4. Discussion

Although models derived from measurements of the different sensors resulted in comparable prediction accuracies (Section 3.2, Table 3 and Table 4), and intercomparison of vegetation indices calculated from UAV and ground-based data indicated that integration of the sensors streams is possible in some cases (Section 3.3, Figure 11), differences could be observed between sensing solutions, especially concerning the first and second data acquisitions (43 and 62 DAP). These differences between datasets can be related to intrinsic sensor characteristics, data spatial support, information sampling and environmental conditions during the measurements. 

Spectral resolution and spectral response of the sensors probably had low impact in the methodology tested here since bands used to derive vegetation indices from UAV and ground-based measurements (Table 2) are comparable (i.e., same or very close bands central wavelengths position and FWHM). Also, reflectance values were derived differently for both sensing approaches. For UAV data a reference panel was used to calculate reflectance factors, as described in Section 2.2, while ground-based reflectance was retrieved through simultaneous acquisition of up-welling radiance and down-welling irradiance, which was measured by a cosine-corrected diffusor [4,8,18]. Despite that, previous studies reported comparable outputs using handheld sensors and reflectance readings calculated through both methods [18], and therefore differences due to this factor are supposed to be small considering the methods evaluated here. 

Ground-based reflectance corresponded to slightly larger footprint (approximately 0.5 m diameter) than the pixel size (approximately 0.2 m, while flying at 80 m height) obtained using UAV measurements. Also spectra were acquired directly over the crop rows at four points inside each plot during ground-based readings (Figure 2), while UAV data represented most of the plot area, after spectra extraction (Figure 12). According to results presented in Section 3.1, Section 3.2 and Section 3.3, these are probably the main factors causing inconsistencies between sensors outputs, due to their impact on the information sampled and changes in the contribution of background or illumination conditions to the reflectance measured. Other authors, comparing ground-based and UAV-carried spectrometers, indicated that a combination of factors, including footprint mismatch (i.e., differences in the area measured), may cause differences in the reflectance values obtained, even if data is acquired in the same date, with comparable sensor footprints and over the same areas [82,83,84]. Conversely, Aasen et al. [85] reported comparable reflectance factors derived from ground-based and UAV measurements corresponding to reference targets, but in this case changes concerning sensor footprint probably have lower impact due to the relative uniformity of these surfaces. 

On the other hand, authors focusing on the calculation of vegetation indices for characterization of crop growth indicate that robust formulations, like NDVI and OSAVI, result in very similar values for data measured in different acquisition levels for the same areas [11,83,86]. Here we demonstrate that some vegetation indices designed to be derived from narrow band spectra, like MCARI/OSAVI_re_ and CI_g_ (Section 3.3, Figure 11), also may provide equivalent outputs between sensor systems for specific period of the growing season, despite differences concerning information sampling, illumination conditions and sun-target-sensor geometry over time. This is an important aspect to consider if one is interested in the integration of different sensing sources using simple approaches like vegetation indices, while benefiting from improved retrieval performance using formulations designed for narrow band spectra. In some cases, even simple linear transfer functions could be used to combine outputs of different sensors, like proposed eventually to integrate data from different satellite systems [13,15], provided that the function itself (i.e., bias from values derived from one sensor in relation to another) is known (e.g., from dataset acquired in previous years) and that the relationship observed is stable over time.

Predictions of crop properties were more accurate for observations acquired latter in the season, after canopy development reached its maximum (Section 3.2, Table 3 and Table 4, Figure 10). Other authors, using ground-based or UAV measurements also arrived to similar conclusions [77,87], indicating that lower background effects and more homogeneous canopy distribution, which are generally characteristics of crops in the middle to late season period, provided better conditions for modelling crop traits. Similar results may also be observed in the assessment of crop properties through RTMs inversion, especially for models including a simplified representation of the vegetation canopy, not taking into account row structure arrangement [88].

Inaccurate crop traits estimation during early season may be detrimental, for instance, to the evaluation of varieties vigor in phenotyping studies [89]. On the other hand, relatively accurate middle to late season retrieval of vegetation properties may still be useful to optimize nitrogen side-dress rates, besides its potential application in diseases assessment and yield estimation [10,90,91,92,93]. Particularly late blight management may benefit from middle to late season monitoring, since climate (i.e., mild-temperatures with high humidity) coupled with canopy closure provide ideal conditions for the pathogen development during this period [72,94,95,96]. In the context of organic production, the most important practices available for in season management of late blight are the application of copper fungicides, which will be banned from 2018 onwards in the European Union, and desiccation of above ground biomass [19,96,97]. Although desiccation using propane burners, after a certain disease incidence threshold, reduces sporangia viability and its spread to nearby fields, it is not advantageous to the producer since yield increases by delaying vine desiccation, due to extra growing days in a period of rapid tubers bulking, are superior than the production loss caused by the pathogen infection until approximately 60% of disease severity [97]. Therefore, early desiccation is mandatorily implemented as national policy to control disease spread, for example in The Netherlands (at approximately 7% of the leaf surface affected), and becomes therefore an important limiting factor for the production. Concerning nitrogen management, organic crops depend mainly on crop rotation and organic fertilization, which are mostly out of season practices [19]. Although few actions are available for in season nutrient management, early within field detection of late blight might allow site-specific control of infection through local use of propane burner or mechanical destruction of affected plants, for example, in areas around the sites affected. In general, crop development monitoring may assist implementation of cultural practices when needed and help to adapt cultivation of future crops considering site-specific characteristics. For instance, intercropping with non-hosts and more resistant varieties, eventually in mixture with others as well, can be alternatives to improve crop protection in organic production of potato, while optical sensing approaches may help to evaluate optimal strip widths for prevention of across strip infection and optimal planting densities of variety mixtures, although these practices need to be evaluated considering different aspects of the crop production (e.g., characteristics of the cultivars to be used, like potential yield and tubers quality; productivity reduction due to smaller surfaces cultivated with the main crop of interest or more productive varieties; etc.) [96,98]. Struik [72] discuss different physiological pathways that can be explored to “escape” late blight infestation, advancing the crop cycle or the tuber bulking, although the author affirms that these practices can only slightly reduce the impact related to disease and indicate that better varieties are a most promising alternative to combat late blight in organic potato cultivation. Lammerts van Bueren et al. [97] indicate that the participation of farmers in the selection of varieties more suitable for organic production can help to complement the comparatively low amount of breeding outputs offered by companies to organic potato producers. In this context, field phenotyping solutions, including optical sensors, can help farmers to better evaluate the varieties under selection. For example, Tiemens-Hulscher et al. [99] evaluated the development of different cultivars, under distinct nitrogen fertilization regimes, based on ground cover estimated through field assessments over the season, an approach that could be adapted to UAV measurements considering the relatively accurate results described here (Table 3 and Table 4). Also, activities related to weed management may benefit from continuous field monitoring in systems under conventional or organic cultivation, despite not being an aspect evaluated in this study. Early or late weed detection in the growing season based on spectral information in the optical domain may be possible and of interested for in season management and out of season cultivation planning [100,101]. Although in this case the sensing effort does not concern directly the crop monitored, weed may have different biochemical and biophysical properties and their development might affect reflectance measurements over an area [102]. Despite that, current research on approaches applied to weed management in agricultural context, especially with UAV data, seem to focus on high spatial resolution imagery and different features extracted from a few spectral bands, while integrating hyperspectral data in monitoring methodologies could provide an increased feature space for infestation detection and intensity assessment, in particular for approaches to be applied later in the growing season [103,104,105].

Best prediction for different crop traits were achieved using different vegetation indices, as presented in Section 3.2 (Table 3 and Table 4). Retrieval of leaf and canopy chlorophyll content was generally more accurate with indices including bands in the red edge, like MTCI and TCARI/OSAVI_re_, or using formulations designed for narrow spectral bands in the visible region in contrast to bands in the near-infrared, like CI_g_. Other studies also report similar results using ground-based, UAV, airborne and satellite derived datasets in comparable scales to the data collected in this study (i.e., at field or plot level) [4,8,12,79,90,106,107,108], indicating that these indices are good options for retrieval and monitoring of leaf and canopy chlorophyll content using simple quantification approaches. More specifically, retrieval based on proximal and remote sensing described by [4,8,12,90] in studies with potato help to confirm trends observed here, concerning leaf and canopy chlorophyll estimations. Conversely, leaf area index was unexpectedly better predicted through indices not especially designed to quantify this trait, like MCARI and OSAVI. This may be related to the fact that the crop canopy was not completely closed at any moment during the growing season (i.e., maximum ground cover of approximately 90%) and leaf area index variation was very small after a certain level of canopy development was reached, probably resulting in low model sensitivity to saturation issues of indices not optimized to leaf area estimation. Also, leaf area index is a difficult trait to estimate in potato even through indirect methods used as ground truth in remote sensing studies (e.g., DHPs, plant canopy analyzer, etc.), due to the large amount of stems and to the canopy configuration. Despite that, R^2^ (0.5–0.6) and RMSE (0.6–0.7 m^2^·m^−2^) obtained using vegetation indices derived from UAV and ground-based reflectance are comparable to those reported in other studies [12,79,87,106,108], including results for potato, and therefore relatively accurate retrieval through indices designed to predict other properties most likely occurred due to specific characteristics of the dataset used in this work. On the other hand, ground cover was generally best predicted using indices sensitive to leaf and canopy properties, i.e., NDVI and OSAVI. The same relatively high accuracy for predictions of this trait using broad band vegetation indices was achieved by other authors [109,110,111]. This can be explained by the relation between fractional cover with reflectance in the whole Vis-NIR spectral region, not only in specific wavelengths, since the effects observed for changes in this property originate mostly from the overall contrast between soil (i.e., background) and vegetation spectral response [112].

Besides good agreement between modelling outputs (Section 3.2, Table 3 and Table 4) and quasi-linear relationship during intercomparison for some vegetation indices (Section 3.3, Figure 11), ground-based and UAV measurements provided comparable descriptive potential concerning the variation of vegetation indices over time, more specifically in relation to late blight development at end of the growing season. This was evaluated in this study through comparison of probability distributions corresponding to vegetation indices derived for the different production systems, as described in Section 3.3 (Figure 13, Table A2). The discriminative potential observed was related mainly to changes in biochemical (i.e., chlorophyll content) and biophysical (i.e., leaf area) properties at canopy level, which were well described by vegetation indices derived from data of the different sensors. Similarly, Nebiker et al. [113] and Whitehead et al. [114] describe the potential for qualitative identification of late blight incidence in potato fields using UAV-based multispectral data with high spatial resolution. Sugiura et al. [91], extended the occurrence detection to severity assessment based also on optical multispectral sensor on board of UAV, and achieved accurate estimates in relation to conventional assessments, even at low incidence levels. These attempts to upscale observations from leaf, as described by Prashar and Jones [115] using thermal data, to canopy level indicate that disease assessment can potentially be performed at field scale, even using multispectral information. Concerning hyperspectral measurements (from 325 to 1075 nm), Ray et al. [116] reported the potential to detect differences between healthy and diseased plants, even at low levels of disease severity (i.e., 0.1%). These results agree with those reported in more frequent studies concerning late blight assessment in tomato (*Solanum lycopersicum*) [117,118,119,120,121,122], although, for this crop, early detection of the disease was reported to be difficult by some authors [120], mainly due to the similarity between the spectral response of diseased and healthy plants.

It is important to notice that in most studies concerning late blight assessment potato traits are not monitored together with disease incidence or severity, and these traits are eventually assumed to be homogeneous before disease occurrence, i.e., comparison of information corresponding to diseased sites with “general” standards of healthy crop, without considering variability in the fields monitored. These facts, indicate that further studies are still needed to better evaluate the suitability of spectral measurements in the optical domain to detect early stages of late blight development in potato, in particular following changes of leaf and canopy traits over time, according to different disease severity levels, in order to allow better description of the disease development and distinction from other stress factors.

## 5. Conclusions

In this study, a comparison of crop trait retrieval through Vis-NIR spectra measured for different acquisition levels was performed. In this respect, results obtained using ground-based and UAV-carried sensors were comparable regardless dissimilarities concerning information sampling and spatial support between them. In the same way, intercomparison of vegetation indices derived from data acquired by the different sensors indicated that, for some indices, results are comparable and could be integrated using even a very simple method, including formulations designed to be derived from narrow spectral bands. Also, spectral data from different acquisition levels presented similar discriminative potential concerning effects of late blight under relatively high infestation severity. These results demonstrate that both sensing solutions tested can deliver accurate outputs, which can be used to model crop traits and follow vegetation growth over time in the context of conventional or organic cropping systems. More specifically, studies exploring sensing technologies applied to organic production are still lacking. Here we verified that even simple modelling techniques coupled with high spatial and spectral resolution information have potential to assist management and phenotyping efforts in organic cropping systems. Although results presented in this article concern estimation of general crop properties related to growth and nutrient status (i.e., leaf chlorophyll content, leaf area index, canopy chlorophyll content and ground cover), specific applications of the monitoring approaches tested need to be further investigated (e.g., early disease detection—specially for late blight, fertilization management, weed assessment, etc.) coupled with more advanced retrieval techniques (e.g., non-parametrical statistical methods, RTMs, etc.), in realistic scenarios. Also, the implementation of integrate monitoring frameworks has to be evaluated concerning accuracy of crop properties estimation and its practical application.

Despite that, general outcomes are positive and indicate a great potential for crop monitoring and phenotyping under organic production scenarios.

## Figures and Tables

**Table 1:** Characteristics of the UAV data collected over time. Days after planting (DAP), estimate potato growth stage (according to the BBCH scale [27,28]), growing degrees day (GDD; using 7 °C as base temperature), flight time (UTC +2), Sun zenith angle (SZA), Sun azimuth angle (SAA), and illumination conditions.

Date	DAP	Growth Stage ^1^	GDD ^2^	Flight Time	SZA ^3^	SAA ^3^	Illum.
05 June 2015	43	3–4	235.7	13:24	29.5	174.5	Clear
24 June 2015	62	4–5	368.9	09:49	52.0	100.4	Overcast
07 July 2015	75	6–7	541.7	10:16	49.0	106.6	Clear
16 July 2015	84	8–9	631.2	10:57	44.3	117.7	Clear
31 July 2015	99	9	774.7	12:15	37.9	144.3	Clear

^1^ BBCH scale: 3—main stem elongation; 4—tuber formation; 5—inflorescence emerging; 6—flowering; 7—fruit development; 8—ripening of fruit and seed; 9—senescence. ^2^ calculated from daily maximum and minimum temperatures from an automated weather station locate at approx. 3 km from the experimental field (weather station “Veenkampen”, 51.9808° N, 5.6217° E); ^3^ SZA and SAA calculated in relation to the center of the images.

**Table 2:** Vegetation indices used to estimate crop traits.

Vegetation Index	Formulation ^1^	Spectral Bands (Centre-nm)		Sensitive to (Scale) ^2^	Ref.
		Ground	UAV		
NDVI	$\frac{R_{800}-R_{670}}{R_{800}+R_{670}}$	670, 780	670, 800	chl, LAI, chl × LAI (L, C)	[68]
WDVI	$R_{870}-{C\;R}_{670};$ $C=\frac{{\mathrm{RSoil}}_{870}}{{\mathrm{RSoil}}_{670}}$	670, 870	670, 870	LAI (C)	[47]
OSAVI	$\left(1+0.16\right)\frac{\left(R_{800}-R_{670}\right)}{\left(R_{800}{+R}_{670}+0.16\right)}$	670, 780	670, 800	chl, LAI, chl × LAI (L, C)	[46]
MCARI	$\left[\left(R_{700}-R_{670}\right)-0.2\left(R_{700}-R_{550}\right)\right]\left(\frac{R_{700}}{R_{670}}\right)$	550, 670, 700	550, 670, 700	chl (L)	[20]
TCARI	$3\left[\left(R_{700}-R_{670}\right)-0.2\left(R_{700}-R_{550}\right)\left(\frac{R_{700}}{R_{670}}\right)\right]$	550, 670, 700	550, 670, 700	chl (L)	[49]
MCARI/OSAVI	$\frac{\left[\left(R_{700}-R_{670}\right)-0.2\left(R_{700}-R_{550}\right)\right]\left(\frac{R_{700}}{R_{670}}\right)}{\left(1+0.16\right)\frac{\left(R_{800}-R_{670}\right)}{\left(R_{800}{+R}_{670}+0.16\right)}}$	550, 670, 700, 780	550, 670, 700, 800	chl (L)	[20]
TCARI/OSAVI	$\frac{3\left[\left(R_{700}-R_{670}\right)-0.2\left(R_{700}-R_{550}\right)\left(\frac{R_{700}}{R_{670}}\right)\right]}{\left(1+0.16\right)\frac{\left(R_{800}-R_{670}\right)}{\left(R_{800}{+R}_{670}+0.16\right)}}$	550, 670, 700, 780	550, 670, 700, 800	chl (L)	[51]
MCARI_re_	$\left[\left(R_{750}-R_{705}\right)-0.2\left(R_{750}-R_{550}\right)\right]\left(\frac{R_{750}}{R_{705}}\right)$	550, 700, 750	550, 705, 750	chl (L)	[52]
MCARI/OSAVI_re_	$\frac{\left[\left(R_{750}-R_{705}\right)-0.2\left(R_{750}-R_{550}\right)\right]\left(\frac{R_{750}}{R_{705}}\right)}{\left(1+0.16\right)\frac{\left(R_{750}-R_{705}\right)}{\left(R_{750}{+R}_{705}+0.16\right)}}$	550, 700, 750	550, 705, 750	chl (L)	[52]
TCARI/OSAVI_re_	$\frac{3\left[\left(R_{750}-R_{705}\right)-0.2\left(R_{750}-R_{550}\right)\left(\frac{R_{750}}{R_{705}}\right)\right]}{\left(1+0.16\right)\frac{\left(R_{750}-R_{705}\right)}{\left(R_{750}{+R}_{705}+0.16\right)}}$	550, 700, 750	550, 705, 750	chl (L)	[52]
CI_re_	$\frac{R_{780}}{R_{710}}-1$	710, 780	710, 780	chl (L)	[54,59]
CI_g_	$\frac{R_{780}}{R_{550}}-1$	710, 750	710, 750	chl (L)	[54,59]
MCARI2	$\frac{1.5\left[2.5\left(R_{800}-R_{670}\right)-1.3\left(R_{800}-R_{550}\right)\right]}{\sqrt{{\left(2R_{800}+1\right)}^{2}-\left(6R_{800}-5\sqrt{R_{670}}\right)-0.5}}$	550, 670, 780	550, 670, 800	LAI (C)	[67]
REP	$700+40\frac{\left(\frac{R_{670}{+R}_{780}}{2}\right)-R_{700}}{R_{740}-R_{700}}$	670, 700, 740, 780	670, 700, 740, 780	chl, LAI, chl × LAI (L, C)	[55]
MTCI	$\frac{R_{754}-R_{709}}{R_{709}-R_{681}}$	670, 710, 750	680, 710, 755	chl, LAI, chl × LAI (L, C)	[57]
PRI	$\frac{R_{570}-R_{531}}{R_{570}{+R}_{531}}$	530, 570	530, 570	xan, car, car/chl, LAI (L, C)	[60]

^1^ R_w_ = reflectance in the spectral band centered in w, RSoil_w_ = reflectance of bare soil in the spectral band centered in w; ^2^ chl = leaf chlorophylls content; LAI = leaf area index; chl × LAI = canopy chlorophylls content; xan = xantophylls; car = carotenoids; car/chl = ratio between carotenoids and chlorophylls; L = leaf scale; C = canopy scale.

**Table 3:** Coefficient of determination and RMSE (from repeated 10-fold cross-validation) for linear regressions between crop traits and vegetation indices calculated from UAV and ground-based data acquired during five data acquisitions (from 43 to 99 DAP; *n* = 60). Indices with best prediction performance for UAV and ground data are indicated in red.

Vegetation Indices	UAV Measurements							
	Leaf Chlorophyll		Leaf Area Index		Canopy Chlorophyll		Ground Cover	
	R^2^	RMSE ^1^	R^2^	RMSE ^1^	R^2^	RMSE ^1^	R^2^	RMSE ^1^
NDVI	0.19	9.42	0.68	0.69	0.39	0.375	0.71	0.069
WDVI	0.00	10.44	0.55	0.81	0.65	0.279	0.74	0.066
OSAVI	0.04	10.24	0.69	0.67	0.58	0.309	0.82	0.055
MCARI	0.37	8.18	0.60	0.78	0.13	0.445	0.44	0.098
TCARI	0.27	8.89	0.70	0.67	0.27	0.407	0.66	0.077
MCARI/OSAVI	0.57	6.63	0.35	0.99	0.00	0.480	0.17	0.120
TCARI/OSAVI	0.63	6.07	0.09	1.19	0.07	0.465	0.02	0.132
MCARI_re_	0.03	10.10	0.31	1.02	0.59	0.303	0.55	0.088
MCARI/OSAVI_re_	0.02	10.18	0.37	0.97	0.63	0.287	0.61	0.082
TCARI/OSAVI_re_	0.03	10.11	0.16	1.12	0.43	0.353	0.35	0.105
CI_re_	0.00	10.37	0.41	0.94	0.61	0.295	0.62	0.081
CI_g_	0.02	10.21	0.47	0.87	0.73	0.241	0.68	0.074
MCARI2	0.03	10.31	0.67	0.69	0.59	0.300	0.81	0.056
REP	0.14	9.40	0.27	1.04	0.68	0.267	0.52	0.092
MTCI	0.19	9.04	0.18	1.11	0.61	0.297	0.41	0.101
PRI	0.00	10.40	0.04	1.21	0.08	0.456	0.11	0.122
	**Ground-Based Measurements**							
NDVI	0.10	9.63	0.41	0.92	0.60	0.295	0.64	0.077
WDVI	0.18	9.11	0.27	1.03	0.58	0.291	0.54	0.087
OSAVI	0.15	9.34	0.35	0.96	0.61	0.288	0.61	0.081
MCARI	0.03	10.22	0.50	0.85	0.34	0.385	0.72	0.068
TCARI	0.01	10.32	0.47	0.88	0.36	0.374	0.72	0.069
MCARI/OSAVI	0.18	9.29	0.43	0.92	0.14	0.441	0.56	0.087
TCARI/OSAVI	0.26	8.81	0.27	1.04	0.04	0.469	0.35	0.104
MCARI_re_	0.19	9.01	0.23	1.06	0.61	0.284	0.49	0.093
MCARI/OSAVI_re_	0.20	8.96	0.24	1.05	0.62	0.279	0.51	0.092
TCARI/OSAVI_re_	0.23	8.75	0.20	1.08	0.62	0.282	0.43	0.099
CI_re_	0.32	8.29	0.21	1.08	0.62	0.281	0.39	0.101
CI_g_	0.44	7.43	0.11	1.15	0.52	0.318	0.26	0.113
MCARI2	0.10	9.65	0.38	0.93	0.61	0.284	0.66	0.076
REP	0.38	7.82	0.07	1.19	0.42	0.360	0.12	0.124
MTCI	0.46	7.25	0.10	1.16	0.53	0.319	0.23	0.116
PRI	0.06	10.04	0.05	1.20	0.01	0.479	0.12	0.124

^1^ RMSE values with same units of corresponding crop properties, i.e., µg·cm^−2^, m^2^·m^−2^ and g·m^−2^ for leaf chlorophyll, leaf area index and canopy chlorophyll, respectively.

**Table 4:** Coefficient of determination and RMSE (from repeated 10-fold cross-validation) for linear regressions between crop traits and vegetation indices calculated from UAV and ground-based data acquired on the last 4 acquisition dates (from 62 to 99 DAP). Only indices with the best prediction performance for both datasets are presented (the complete results are shown in the Table A1).

Traits	UAV Measurements		
	Vegetation Index	R^2^	RMSE ^1^
Leaf chlorophyll	MTCI	0.91	2.31
Leaf area index	TCARI	0.56	0.68
Canopy chlorophyll	CI_g_	0.81	0.201
Ground cover	NDVI	0.90	0.037
	**Ground-Based Measurements**		
Leaf chlorophyll	TCARI/OSAVI_re_	0.78	3.52
Leaf area index	OSAVI	0.58	0.63
Canopy chlorophyll	CI_g_	0.83	0.194
Ground cover	OSAVI	0.92	0.033

^1^ RMSE values with same units of corresponding crop properties, i.e., µg·cm^−2^, m^2^·m^−2^ and g·m^−2^ for leaf chlorophyll, leaf area index and canopy chlorophyll, respectively.

## Appendix A

**Table A1:** Coefficient of determination and RMSE (from repeated 10-fold cross-validation) for linear regressions between crop traits and vegetation indices calculated from UAV and ground-based data acquired on the last four acquisition dates (from 62 to 99 DAP). Indices with best prediction performance for UAV and ground-based measurements are indicated in red (*n* = 60).

Vegetation Indices	UAV Measurements							
	Leaf Chlorophyll		Leaf Area Index		Canopy Chlorophyll		Ground Cover	
	R^2^	RMSE ^1^	R^2^	RMSE ^1^	R^2^	RMSE ^1^	R^2^	RMSE ^1^
NDVI	0.38	6.03	0.62	0.60	0.74	0.239	0.90	0.037
WDVI	0.66	4.36	0.36	0.82	0.75	0.237	0.67	0.066
OSAVI	0.53	5.24	0.53	0.69	0.80	0.208	0.85	0.046
MCARI	0.08	7.33	0.38	0.81	0.05	0.471	0.22	0.102
TCARI	0.00	7.56	0.56	0.68	0.26	0.414	0.56	0.079
MCARI/OSAVI	0.42	5.77	0.12	0.94	0.03	0.471	0.01	0.111
TCARI/OSAVI	0.71	4.01	0.00	0.99	0.23	0.406	0.04	0.106
MCARI_re_	0.82	3.22	0.14	0.94	0.61	0.298	0.46	0.084
MCARI/OSAVI_re_	0.80	3.35	0.19	0.91	0.66	0.275	0.53	0.079
TCARI/OSAVI_re_	0.86	2.82	0.02	0.98	0.42	0.354	0.21	0.096
CI_re_	0.87	2.80	0.18	0.93	0.68	0.270	0.50	0.081
CI_g_	0.80	3.42	0.33	0.83	0.81	0.201	0.63	0.071
MCARI2	0.53	5.21	0.49	0.72	0.77	0.223	0.81	0.051
REP	0.88	2.66	0.21	0.90	0.72	0.249	0.54	0.078
MTCI	0.91	2.31	0.14	0.94	0.65	0.281	0.44	0.085
PRI	0.28	6.49	0.03	0.96	0.04	0.469	0.02	0.109
	**Ground-Based Measurements**							
NDVI	0.33	6.18	0.63	0.59	0.70	0.255	0.92	0.032
WDVI	0.52	5.20	0.44	0.75	0.72	0.240	0.82	0.050
OSAVI	0.41	5.83	0.58	0.63	0.74	0.233	0.92	0.033
MCARI	0.10	7.16	0.37	0.81	0.30	0.400	0.69	0.064
TCARI	0.13	7.00	0.37	0.80	0.34	0.386	0.71	0.062
MCARI/OSAVI	0.00	7.56	0.20	0.90	0.07	0.467	0.40	0.090
TCARI/OSAVI	0.03	7.46	0.04	0.98	0.00	0.481	0.15	0.105
MCARI_re_	0.70	4.11	0.31	0.84	0.72	0.243	0.66	0.067
MCARI/OSAVI_re_	0.67	4.28	0.34	0.82	0.74	0.232	0.70	0.063
TCARI/OSAVI_re_	0.78	3.52	0.27	0.85	0.74	0.237	0.57	0.074
CI_re_	0.65	4.53	0.45	0.74	0.83	0.194	0.72	0.061
CI_g_	0.67	4.33	0.43	0.75	0.83	0.194	0.67	0.067
MCARI2	0.42	5.75	0.53	0.68	0.71	0.244	0.89	0.038
REP	0.60	4.76	0.27	0.85	0.65	0.282	0.35	0.092
MTCI	0.69	4.18	0.41	0.76	0.83	0.196	0.64	0.070
PRI	0.00	7.64	0.00	0.99	0.00	0.482	0.05	0.110

^1^ RMSE values with same units of corresponding crop properties, i.e., µg·cm^−2^, m^2^·m^−2^ and g·m^−2^ for leaf chlorophyll, leaf area index and canopy chlorophyll, respectively.

**Table A2:** Bhattacharyya coefficient (B-coefficient) between distributions of vegetation indices, calculated from UAV and ground-based measurements, corresponding to each production system (i.e., non-mixed and mixed varieties). Only values calculated for the two last data acquisitions (84 and 99 DAP) are presented. Indices giving the best treatments distinction (i.e., largest B-coefficients) for each sensor system are indicated in red.

Vegetation Indices	B-Coefficient				Differ. between B-Coeff. (99 DAP–84 DAP)	
	84 DAP		99 DAP			
	UAV	Ground	UAV	Ground	UAV	Ground
NDVI	0.168	0.271	0.191	0.236	0.023	−0.035
WDVI	0.353	0.343	0.38	0.323	0.027	−0.020
OSAVI	0.202	0.309	0.282	0.251	0.080	−0.058
MCARI	0.374	0.371	0.457	0.388	0.083	0.017
TCARI	0.332	0.339	0.373	0.32	0.041	−0.019
MCARI/OSAVI	0.357	0.394	0.429	0.351	0.072	−0.043
TCARI/OSAVI	0.367	0.364	0.420	0.325	0.053	−0.039
MCARI_re_	0.385	0.405	0.517	0.451	0.132	0.046
MCARI/OSAVI_re_	0.359	0.395	0.393	0.407	0.034	0.012
TCARI/OSAVI_re_	0.363	0.375	0.182	0.232	−0.181	−0.143
CI_re_	0.428	0.427	0.479	0.49	0.051	0.063
CI_g_	0.522	0.428	0.511	0.492	−0.011	0.064
MCARI2	0.258	0.301	0.358	0.319	0.100	0.018
REP	0.460	0.385	0.420	0.446	−0.040	0.061
MTCI	0.445	0.427	0.457	0.511	0.012	0.084
PRI	0.333	0.464	0.321	0.404	−0.012	−0.060

## References

[1] Mulla D.J. (2013). Twenty five years of remote sensing in precision agriculture: Key advances and remaining knowledge gaps. Biosyst. Eng..

[2] Yost M.A., Kitchen N.R., Sudduth K.A., Sadler E.J., Drummond S.T., Volkmann M.R. (2016). Long-term impact of a precision agriculture system on grain crop production. Precis. Agric..

[3] Sankaran S., Khot L.R., Espinoza C.Z., Jarolmasjed S., Sathuvalli V.R., Vandemark G.J., Miklas P.N., Carter A.H., Pumphrey M.O., Knowles N.R. (2015). Low-altitude, high-resolution aerial imaging systems for row and field crop phenotyping: A review. Eur. J. Agron..

[4] Clevers J.G.P.W., Kooistra L. (2012). Using Hyperspectral Remote Sensing Data for Retrieving Canopy Chlorophyll and Nitrogen Content. IEEE J. Sel. Top. Appl. Earth Obs. Remote Sens..

[5] Li L., Zhang Q., Huang D. (2014). A Review of Imaging Techniques for Plant Phenotyping. Sensors.

[6] Verrelst J., Camps-Valls G., Muñoz-Marí J., Rivera J.P., Veroustraete F., Clevers J.G.P.W., Moreno J. (2015). Optical remote sensing and the retrieval of terrestrial vegetation bio-geophysical properties—A review. ISPRS J. Photogramm. Remote Sens..

[7] Atzberger C., Darvishzadeh R., Immitzer M., Schlerf M., Skidmore A., le Maire G. (2015). Comparative analysis of different retrieval methods for mapping grassland leaf area index using airborne imaging spectroscopy. Int. J. Appl. Earth Obs. Geoinf..

[8] Kooistra L., Clevers J.G.P.W. (2016). Estimating potato leaf chlorophyll content using ratio vegetation indices. Remote Sens. Lett..

[9] Mahlein A.-K., Rumpf T., Welke P., Dehne H.-W., Plümer L., Steiner U., Oerke E.-C. (2013). Development of spectral indices for detecting and identifying plant diseases. Remote Sens. Environ..

[10] Tanger P., Klassen S., Mojica J.P., Lovell J.T., Moyers B.T., Baraoidan M., Naredo M.E.B., McNally K.L., Poland J., Bush D.R. (2017). Field-based high throughput phenotyping rapidly identifies genomic regions controlling yield components in rice. Sci. Rep..

[11] Zaman-Allah M., Vergara O., Araus J.L., Tarekegne A., Magorokosho C., Zarco-Tejada P.J., Hornero A., Albà A.H., Das B., Craufurd P. (2015). Unmanned aerial platform-based multi-spectral imaging for field phenotyping of maize. Plant Methods.

[12] Clevers J.G.P.W., Kooistra L., van den Brande M.M.M. (2017). Using Sentinel-2 Data for Retrieving LAI and Leaf and Canopy Chlorophyll Content of a Potato Crop. Remote Sens..

[13] Steven M.D., Malthus T.J., Baret F., Xu H., Chopping M.J. (2003). Intercalibration of vegetation indices from different sensor systems. Remote Sens. Environ..

[14] Kim Y., Huete A.R., Miura T., Jiang Z. (2010). Spectral compatibility of vegetation indices across sensors: Band decomposition analysis with Hyperion data. J. Appl. Remote Sens..

[15] Chander G., Hewison T.J., Fox N., Wu X., Xiong X., Blackwell W.J. (2013). Overview of intercalibration of satellite instruments. IEEE Trans. Geosci. Remote Sens..

[16] Zhang H., Huang B. (2013). Support Vector Regression-Based Downscaling for Intercalibration of Multiresolution Satellite Images. IEEE Trans. Geosci. Remote Sens..

[17] Fitzgerald G.J. (2010). Characterizing vegetation indices derived from active and passive sensors. Int. J. Remote Sens..

[18] Yao X., Yao X., Jia W., Tian Y., Ni J., Cao W., Zhu Y. (2013). Comparison and Intercalibration of Vegetation Indices from Different Sensors for Monitoring Above-Ground Plant Nitrogen Uptake in Winter Wheat. Sensors.

[19] Finckh M.R., Schulte-Geldermann E., Bruns C. (2006). Challenges to Organic Potato Farming: Disease and Nutrient Management. Potato Res..

[20] Daughtry C.S.T., Walthall C.L., Kim M.S., De Colstoun E.B., McMurtrey J.E. (2000). Estimating corn leaf chlorophyll concentration from leaf and canopy reflectance. Remote Sens. Environ..

[21] Suárez L., Zarco-Tejada P.J., Sepulcre-Cantó G., Pérez-Priego O., Miller J.R., Jiménez-Muñoz J.C., Sobrino J. (2008). Assessing canopy PRI for water stress detection with diurnal airborne imagery. Remote Sens. Environ..

[22] Ammann K. (2009). Why farming with high tech methods should integrate elements of organic agriculture. New Biotechnol..

[23] European and Mediterranean Plant Protection Organization (2008). Phytophthora infestans on potato. EPPO Bull..

[24] Suomalainen J., Anders N., Iqbal S., Roerink G., Franke J., Wenting P., Hünniger D., Bartholomeus H., Becker R., Kooistra L. (2014). A Lightweight Hyperspectral Mapping System and Photogrammetric Processing Chain for Unmanned Aerial Vehicles. Remote Sens..

[25] Schläpfer D., Richter R. (2002). Geo-atmospheric processing of airborne imaging spectrometry data. Part 1: Parametric orthorectification. Int. J. Remote Sens..

[26] Ullman S. (1979). The Interpretation of Structure from Motion. Proc. R. Soc. Lond. B Biol. Sci..

[27] Lancashire P.D., Bleiholder H., Boom T.V.D., Langelüddeke P., Stauss R., Weber E., Witzenberger A. (1991). A uniform decimal code for growth stages of crops and weeds. Ann. Appl. Biol..

[28] Jki O. (2010). Growth stages of mono-and dicotyledonous plants. BBCH Scale.

[29] Habib A., Xiong W., He F., Yang H.L., Crawford M. (2017). Improving Orthorectification of UAV-Based Push-Broom Scanner Imagery Using Derived Orthophotos From Frame Cameras. IEEE J. Sel. Top. Appl. Earth Obs. Remote Sens..

[30] Canny J. (1986). A computational approach to edge detection. IEEE Trans. Pattern Anal. Mach. Intell..

[31] Uddling J., Gelang-Alfredsson J., Piikki K., Pleijel H. (2007). Evaluating the relationship between leaf chlorophyll concentration and SPAD-502 chlorophyll meter readings. Photosynth. Res..

[32] Parry C., Blonquist J.M., Bugbee B. (2014). In situ measurement of leaf chlorophyll concentration: Analysis of the optical/absolute relationship: The optical/absolute chlorophyll relationship. Plant Cell Environ..

[33] Darvishzadeh R., Skidmore A., Schlerf M., Atzberger C. (2008). Inversion of a radiative transfer model for estimating vegetation LAI and chlorophyll in a heterogeneous grassland. Remote Sens. Environ..

[34] Weiss M., Baret F., Smith G.J., Jonckheere I., Coppin P. (2004). Review of methods for in situ leaf area index (LAI) determination. Agric. For. Meteorol..

[35] Demarez V., Duthoit S., Baret F., Weiss M., Dedieu G. (2008). Estimation of leaf area and clumping indexes of crops with hemispherical photographs. Agric. For. Meteorol..

[36] Baret F. A Simple Method to Calibrate Hemispherical Photographs.

[37] Liu Y., Mu X., Wang H., Yan G. (2012). A novel method for extracting green fractional vegetation cover from digital images. J. Veg. Sci..

[38] Song W., Mu X., Yan G., Huang S. (2015). Extracting the Green Fractional Vegetation Cover from Digital Images Using a Shadow-Resistant Algorithm (SHAR-LABFVC). Remote Sens..

[39] Nilson T. (1971). A theoretical analysis of the frequency of gaps in plant stands. Agric. Meteorol..

[40] Garrigues S., Shabanov N.V., Swanson K., Morisette J.T., Baret F., Myneni R.B. (2008). Intercomparison and sensitivity analysis of Leaf Area Index retrievals from LAI-2000, AccuPAR, and digital hemispherical photography over croplands. Agric. For. Meteorol..

[41] Campbell G.S. (1990). Derivation of an angle density function for canopies with ellipsoidal leaf angle distributions. Agric. For. Meteorol..

[42] Bréda N.J.J. (2003). Ground-based measurements of leaf area index: A review of methods, instruments and current controversies. J. Exp. Bot..

[43] Lang A.R.G., Xiang Y. (1986). Estimation of leaf area index from transmission of direct sunlight in discontinuous canopies. Agric. For. Meteorol..

[44] Van Gardingen P.R., Jackson G.E., Hernandez-Daumas S., Russell G., Sharp L. (1999). Leaf area index estimates obtained for clumped canopies using hemispherical photography. Agric. For. Meteorol..

[45] Jonckheere I., Fleck S., Nackaerts K., Muys B., Coppin P., Weiss M., Baret F. (2004). Review of methods for in situ leaf area index determination: Part I. Theories, sensors and hemispherical photography. Agric. For. Meteorol..

[46] Rondeaux G., Steven M., Baret F. (1996). Optimization of soil-adjusted vegetation indices. Remote Sens. Environ..

[47] Clevers J. (1988). The derivation of a simplified reflectance model for the estimation of leaf area index. Remote Sens. Environ..

[48] Clevers J.G.P.W., Manakos I., Braun M. (2014). Beyond NDVI: Extraction of Biophysical Variables from Remote Sensing Imagery. Land Use and Land Cover Mapping in Europe.

[49] Broge N.H., Leblanc E. (2001). Comparing prediction power and stability of broadband and hyperspectral vegetation indices for estimation of green leaf area index and canopy chlorophyll density. Remote Sens. Environ..

[50] Kim M.S., Daughtry C.S.T., Chappelle E.W., Mcmurtrey J.E., Walthall C.L. The use of high spectral resolution bands for estimating absorbed photosynthetically active radiation (Apar). Proceedings of the Sixth Symposium on Physical Measurements and Signatures in Remote Sensing.

[51] Haboudane D., Miller J.R., Tremblay N., Zarco-Tejada P.J., Dextraze L. (2002). Integrated narrow-band vegetation indices for prediction of crop chlorophyll content for application to precision agriculture. Remote Sens. Environ..

[52] Wu C., Niu Z., Tang Q., Huang W. (2008). Estimating chlorophyll content from hyperspectral vegetation indices: Modeling and validation. Agric. For. Meteorol..

[53] Ciganda V., Gitelson A., Schepers J. (2009). Non-destructive determination of maize leaf and canopy chlorophyll content. J. Plant Physiol..

[54] Gitelson A.A., Gritz Y., Merzlyak M.N. (2003). Relationships between leaf chlorophyll content and spectral reflectance and algorithms for non-destructive chlorophyll assessment in higher plant leaves. J. Plant Physiol..

[55] Guyot G., Baret F. Utilisation de la haute resolution spectrale pour suivre l’etat des couverts vegetaux. Proceedings of the 4th International Colloquium on Spectral Signatures of Objects in Remote Sensing.

[56] Cho M.A., Skidmore A.K. (2006). A new technique for extracting the red edge position from hyperspectral data: The linear extrapolation method. Remote Sens. Environ..

[57] Dash J., Curran P.J. (2004). The MERIS terrestrial chlorophyll index. Int. J. Remote Sens..

[58] Van der Meij B., Kooistra L., Suomalainen J., Barel J.M., De Deyn G.B. (2017). Remote sensing of plant trait responses to field-based plant–soil feedback using UAV-based optical sensors. Biogeosciences..

[59] Gitelson A.A., Keydan G.P., Merzlyak M.N. (2006). Three-band model for noninvasive estimation of chlorophyll, carotenoids, and anthocyanin contents in higher plant leaves. Geophys. Res. Lett..

[60] Gamon J.A., Penuelas J., Field C.B. (1992). A narrow-waveband spectral index that tracks diurnal changes in photosynthetic efficiency. Remote Sens. Environ..

[61] Sims D.A., Gamon J.A. (2002). Relationships between leaf pigment content and spectral reflectance across a wide range of species, leaf structures and developmental stages. Remote Sens. Environ..

[62] Ustin S.L., Gitelson A.A., Jacquemoud S., Schaepman M., Asner G.P., Gamon J.A., Zarco-Tejada P. (2009). Retrieval of foliar information about plant pigment systems from high resolution spectroscopy. Remote Sens. Environ..

[63] Garrity S.R., Eitel J.U.H., Vierling L.A. (2011). Disentangling the relationships between plant pigments and the photochemical reflectance index reveals a new approach for remote estimation of carotenoid content. Remote Sens. Environ..

[64] Gitelson A.A., Gamon J.A., Solovchenko A. (2017). Multiple drivers of seasonal change in PRI: Implications for photosynthesis 1. Leaf level. Remote Sens. Environ..

[65] Suárez L., Zarco-Tejada P.J., Berni J.A.J., González-Dugo V., Fereres E. (2009). Modelling PRI for water stress detection using radiative transfer models. Remote Sens. Environ..

[66] Gitelson A.A., Gamon J.A., Solovchenko A. (2017). Multiple drivers of seasonal change in PRI: Implications for photosynthesis 2. Stand level. Remote Sens. Environ..

[67] Haboudane D. (2004). Hyperspectral vegetation indices and novel algorithms for predicting green LAI of crop canopies: Modeling and validation in the context of precision agriculture. Remote Sens. Environ..

[68] Rouse J., Haas R.H., Schell J.A., Deering D.W. Monitoring vegetation systems in the Great Plains with ERTS. Proceedings of the Third Earth Resources Technology Satellite-1 Symposium.

[69] Inoue Y., Guérif M., Baret F., Skidmore A., Gitelson A., Schlerf M., Darvishzadeh R., Olioso A. (2016). Simple and robust methods for remote sensing of canopy chlorophyll content: A comparative analysis of hyperspectral data for different types of vegetation: Simple sensing of canopy chlorophyll content. Plant Cell Environ..

[70] Bhattacharyya A. (1946). On a Measure of Divergence between Two Multinomial Populations. Sankhyā Indian J. Stat. (1933–1960).

[71] Goffart J.P., Olivier M., Frankinet M. (2008). Potato Crop Nitrogen Status Assessment to Improve N Fertilization Management and Efficiency: Past–Present–Future. Potato Res..

[72] Struik P.C. (2010). Can Physiology Help Us to Combat Late Blight in Potato?. Potato Res..

[73] Gitelson A.A., Peng Y., Arkebauer T.J., Schepers J. (2014). Relationships between gross primary production, green LAI, and canopy chlorophyll content in maize: Implications for remote sensing of primary production. Remote Sens. Environ..

[74] Kimes D.S. (1983). Dynamics of directional reflectance factor distributions for vegetation canopies. Appl. Opt..

[75] Hatfield J.L., Gitelson A.A., Schepers J.S., Walthall C.L. (2008). Application of Spectral Remote Sensing for Agronomic Decisions. Agron. J..

[76] Haboudane D., Tremblay N., Miller J.R., Vigneault P. (2008). Remote Estimation of Crop Chlorophyll Content Using Spectral Indices Derived from Hyperspectral Data. IEEE Trans. Geosci. Remote Sens..

[77] Yu K., Lenz-Wiedemann V., Chen X., Bareth G. (2014). Estimating leaf chlorophyll of barley at different growth stages using spectral indices to reduce soil background and canopy structure effects. ISPRS J. Photogramm. Remote Sens..

[78] Gitelson A.A., Viña A., Ciganda V., Rundquist D.C., Arkebauer T.J. (2005). Remote estimation of canopy chlorophyll content in crops. Geophys. Res. Lett..

[79] Viña A., Gitelson A.A., Nguy-Robertson A.L., Peng Y. (2011). Comparison of different vegetation indices for the remote assessment of green leaf area index of crops. Remote Sens. Environ..

[80] Hruska R., Mitchell J., Anderson M., Glenn N.F. (2012). Radiometric and Geometric Analysis of Hyperspectral Imagery Acquired from an Unmanned Aerial Vehicle. Remote Sens..

[81] Habib A., Han Y., Xiong W., He F., Zhang Z., Crawford M. (2016). Automated Ortho-Rectification of UAV-Based Hyperspectral Data over an Agricultural Field Using Frame RGB Imagery. Remote Sens..

[82] Von Bueren S.K., Burkart A., Hueni A., Rascher U., Tuohy M.P., Yule I.J. (2015). Deploying four optical UAV-based sensors over grassland: Challenges and limitations. Biogeosciences..

[83] Bareth G., Aasen H., Bendig J., Gnyp M.L., Bolten A., Jung A., Michels R., Soukkamäki J. (2015). Low-weight and UAV-based hyperspectral full-frame cameras for monitoring crops: Spectral comparison with portable spectroradiometer measurements. Photogramm. Fernerkund. Geoinf..

[84] Jakob S., Zimmermann R., Gloaguen R. (2017). The Need for Accurate Geometric and Radiometric Corrections of Drone-Borne Hyperspectral Data for Mineral Exploration: MEPHySTo—A Toolbox for Pre-Processing Drone-Borne Hyperspectral Data. Remote Sens..

[85] Aasen H., Burkart A., Bolten A., Bareth G. (2015). Generating 3D hyperspectral information with lightweight UAV snapshot cameras for vegetation monitoring: From camera calibration to quality assurance. ISPRS J. Photogramm. Remote Sens..

[86] Tattaris M., Reynolds M.P., Chapman S.C. (2016). A Direct Comparison of Remote Sensing Approaches for High-Throughput Phenotyping in Plant Breeding. Front. Plant Sci..

[87] Lelong C.C.D., Burger P., Jubelin G., Roux B., Labbé S., Baret F. (2008). Assessment of Unmanned Aerial Vehicles Imagery for Quantitative Monitoring of Wheat Crop in Small Plots. Sensors.

[88] Dorigo W.A. (2012). Improving the Robustness of Cotton Status Characterisation by Radiative Transfer Model Inversion of Multi-Angular CHRIS/PROBA Data. IEEE J. Sel. Top. Appl. Earth Obs. Remote Sens..

[89] Cowley R.B., Luckett D.J., Moroni J.S., Diffey S. (2014). Use of remote sensing to determine the relationship of early vigour to grain yield in canola (Brassica napus L.) germplasm. Crop Pasture Sci..

[90] Nigon T.J., Mulla D.J., Rosen C.J., Cohen Y., Alchanatis V., Knight J., Rud R. (2015). Hyperspectral aerial imagery for detecting nitrogen stress in two potato cultivars. Comput. Electron. Agric..

[91] Sugiura R., Tsuda S., Tamiya S., Itoh A., Nishiwaki K., Murakami N., Shibuya Y., Hirafuji M., Nuske S. (2016). Field phenotyping system for the assessment of potato late blight resistance using RGB imagery from an unmanned aerial vehicle. Biosyst. Eng..

[92] Vergara-Díaz O., Zaman-Allah M.A., Masuka B., Hornero A., Zarco-Tejada P., Prasanna B.M., Cairns J.E., Araus J.L. (2016). A Novel Remote Sensing Approach for Prediction of Maize Yield Under Different Conditions of Nitrogen Fertilization. Front. Plant Sci..

[93] Van Evert F.K., Booij R., Jukema J.N., ten Berge H.F.M., Uenk D., Meurs E.J.J. (Bert), van Geel W.C.A., Wijnholds K.H., Slabbekoorn J.J. (Hanja) (2012). Using crop reflectance to determine sidedress N rate in potato saves N and maintains yield. Eur. J. Agron..

[94] Johnson D.A., Hamm P.B., Miller J.S., Porter L.D., He Z., Larkin R., Honeycutt W. (2012). Late Blight Epidemics in the Columbia Basin. Sustainable Potato Production: Global Case Studies.

[95] Termorshuizen A.J. (2007). Chapter 29—Fungal and Fungus-Like Pathogens of Potato. Potato Biology and Biotechnology.

[96] Cooke L.R., Schepers H.T.A.M., Hermansen A., Bain R.A., Bradshaw N.J., Ritchie F., Shaw D.S., Evenhuis A., Kessel G.J.T., Wander J.G.N. (2011). Epidemiology and Integrated Control of Potato Late Blight in Europe. Potato Res..

[97] Lammerts van Bueren E.T., Tiemens-Hulscher M., Struik P.C. (2008). Cisgenesis Does Not Solve the Late Blight Problem of Organic Potato Production: Alternative Breeding Strategies. Potato Res..

[98] Bouws H., Finckh M.R. (2008). Effects of strip intercropping of potatoes with non-hosts on late blight severity and tuber yield in organic production. Plant Pathol..

[99] Tiemens-Hulscher M., van Bueren E.T.L., Struik P.C. (2014). Identifying nitrogen-efficient potato cultivars for organic farming. Euphytica.

[100] Melander B., Rasmussen I.A., Bàrberi P. (2005). Integrating physical and cultural methods of weed control—Examples from European research. Weed Sci..

[101] López-Granados F. (2011). Weed detection for site-specific weed management: Mapping and real-time approaches: Weed detection for site-specific weed management. Weed Res..

[102] López-Granados F., Peña-Barragán J.M., Jurado-Expósito M., Francisco-Fernández M., Cao R., Alonso-Betanzos A., Fontenla-Romero O. (2008). Multispectral classification of grass weeds and wheat (Triticum durum) using linear and nonparametric functional discriminant analysis and neural networks. Weed Res..

[103] Hung C., Xu Z., Sukkarieh S. (2014). Feature Learning Based Approach for Weed Classification Using High Resolution Aerial Images from a Digital Camera Mounted on a UAV. Remote Sens..

[104] Behmann J., Mahlein A.-K., Rumpf T., Römer C., Plümer L. (2015). A review of advanced machine learning methods for the detection of biotic stress in precision crop protection. Precis. Agric..

[105] Pérez-Ortiz M., Peña J.M., Gutiérrez P.A., Torres-Sánchez J., Hervás-Martínez C., López-Granados F. (2016). Selecting patterns and features for between- and within- crop-row weed mapping using UAV-imagery. Expert Syst. Appl..

[106] Berni J., Zarco-Tejada P.J., Suarez L., Fereres E. (2009). Thermal and Narrowband Multispectral Remote Sensing for Vegetation Monitoring From an Unmanned Aerial Vehicle. IEEE Trans. Geosci. Remote Sens..

[107] Hunt E.R., Doraiswamy P.C., McMurtrey J.E., Daughtry C.S.T., Perry E.M., Akhmedov B. (2013). A visible band index for remote sensing leaf chlorophyll content at the canopy scale. Int. J. Appl. Earth Obs. Geoinf..

[108] Zarco-Tejada P.J., Morales A., Testi L., Villalobos F.J. (2013). Spatio-temporal patterns of chlorophyll fluorescence and physiological and structural indices acquired from hyperspectral imagery as compared with carbon fluxes measured with eddy covariance. Remote Sens. Environ..

[109] Mesas-Carrascosa F.-J., Torres-Sánchez J., Clavero-Rumbao I., García-Ferrer A., Peña J.-M., Borra-Serrano I., López-Granados F. (2015). Assessing Optimal Flight Parameters for Generating Accurate Multispectral Orthomosaicks by UAV to Support Site-Specific Crop Management. Remote Sens..

[110] Imukova K., Ingwersen J., Streck T. (2015). Determining the spatial and temporal dynamics of the green vegetation fraction of croplands using high-resolution RapidEye satellite images. Agric. For. Meteorol..

[111] Svensgaard J., Roitsch T., Christensen S. (2014). Development of a Mobile Multispectral Imaging Platform for Precise Field Phenotyping. Agronomy.

[112] Verrelst J., Alonso L., Camps-Valls G., Delegido J., Moreno J. (2012). Retrieval of Vegetation Biophysical Parameters Using Gaussian Process Techniques. IEEE Trans. Geosci. Remote Sens..

[113] Nebiker S., Lack N., Abächerli M., Läderach S. (Volume XLI-B1). Light-weight multispectral UAV sensors and their capabilities for predicting grain yield and detecting plant diseases. Proceedings of the ISPRS—International Archives of the Photogrammetry, Remote Sensing and Spatial Information Sciences.

[114] Whitehead K., Hugenholtz C.H., Myshak S., Brown O., LeClair A., Tamminga A., Barchyn T.E., Moorman B., Eaton B. (2014). Remote sensing of the environment with small unmanned aircraft systems (UASs), part 2: Scientific and commercial applications. J. Unmanned Veh. Syst..

[115] Prashar A., Jones H.G. (2014). Infra-Red Thermography as a High-Throughput Tool for Field Phenotyping. Agronomy.

[116] Ray S.S., Jain N., Arora R.K., Chavan S., Panigrahy S. (2011). Utility of Hyperspectral Data for Potato Late Blight Disease Detection. J. Indian Soc. Remote Sens..

[117] Zhang M., Liu X., O’Neill M. (2002). Spectral discrimination of Phytophthora infestans infection on tomatoes based on principal component and cluster analyses. Int. J. Remote Sens..

[118] Zhang M., Qin Z., Liu X., Ustin S.L. (2003). Detection of stress in tomatoes induced by late blight disease in California, USA, using hyperspectral remote sensing. Int. J. Appl. Earth Obs. Geoinf..

[119] Zhang M., Qin Z. Spectral analysis of tomato late blight infections for remote sensing of tomato disease stress in California. Proceedings of the 2004 IEEE International Geoscience and Remote Sensing Symposium (IGARSS’04).

[120] Zhang M., Qin Z., Liu X. (2005). Remote Sensed Spectral Imagery to Detect Late Blight in Field Tomatoes. Precis. Agric..

[121] Wang X., Zhang M., Zhu J., Geng S. (2008). Spectral prediction of Phytophthora infestans infection on tomatoes using artificial neural network (ANN). Int. J. Remote Sens..

[122] Xie C., Shao Y., Li X., He Y. (2015). Detection of early blight and late blight diseases on tomato leaves using hyperspectral imaging. Sci. Rep..

