# Correction: Domingues Franceschini, M.H.; et al. Intercomparison of Unmanned Aerial Vehicle and Ground-Based Narrow Band Spectrometers Applied to Crop Trait Monitoring in Organic Potato Production. *Sensors* 2017, *17*, 1428

**DOI:** 10.3390/s17102265

**Published:** 2017-10-02

**Authors:** Marston Héracles Domingues Franceschini, Harm Bartholomeus, Dirk van Apeldoorn, Juha Suomalainen, Lammert Kooistra

**Affiliations:** 1 Laboratory of Geo-Information Science and Remote Sensing, Wageningen University and Research, P.O. Box 47, 6700 AA Wageningen, The Netherlands; harm.bartholomeus@wur.nl (H.B.); juha.suomalainen@nls.fi (J.S.); lammert.kooistra@wur.nl (L.K.); 2 Farming Systems Ecology group, Wageningen University and Research, P.O. Box 430, 6700 AK Wageningen, The Netherlands; dirk.vanapeldoorn@wur.nl; 3 Finnish Geospatial Research Institute, National Land Survey of Finland, Geodeetinrinne 1, 02430 Masala, Finland

The authors would like to correct Figure 13 and Table A2, as well as the text related to the data presented in both of them, as indicated below, considering that an error in the calculations involving Equation (2), described in the Section 2.8 of the Materials and Methods Section, resulted in the communication of incorrect values [1]. Despite that, the conclusions of the article continue the same. The authors apologize for any inconvenience caused to the readers by this error. 

## 3.3. Intercomparison of UAV and Ground-Based Spectra

The vegetation index providing the best discriminative potential between treatments for UAV and ground-based measurements, MCARI2 (Modified Chlorophyll Absorption Ratio Index 2, Table 2), yielded good estimates of canopy properties, especially for chlorophyll content, leaf area, and ground cover (Tables 3 and A1). This indicates that indices describing not only leaf properties but also canopy traits resulted in better segregation of crops with this specific late blight incidence level, in relation to relatively healthier plants. Plots with mixed varieties presented disease severity varying between 25% and 75% of leaf area dead per plot, on the last acquisition date. At this infection stage, not only leaf biochemical composition was affected by the pathogen development, but also structural properties at the canopy level, and therefore indices describing global canopy health status were more effective for treatments segregation.

## Figures and Tables

**Figure 13:**
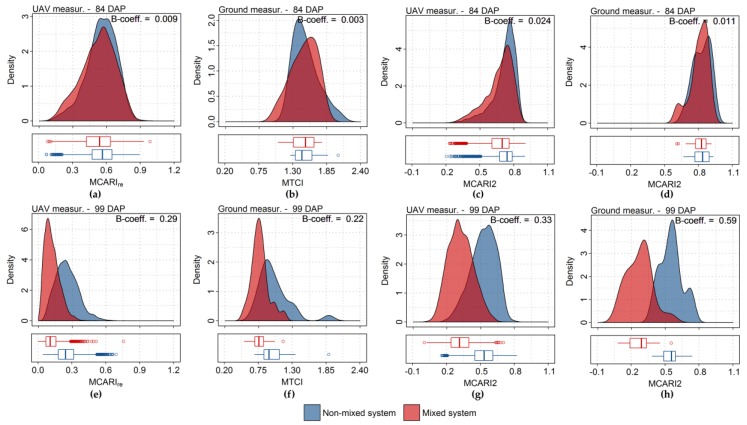
Distribution of MCARIre (Modified Chlorophyll Absorption Ratio Index Red Edge, Table 2), MTCI (MERIS terrestrial chlorophyll index, Table 2), and MCARI2 calculated from UAV (**a**,**c**,**e**,**g**) and ground-based data (**b**,**d**,**f**,**h**), respectively. Estimate probability density and boxplot corresponding to observations from a given sensor and acquisition date are presented for each treatment (i.e., non-mixed and mixed systems). Results concerning UAV and ground data correspond, respectively, to approximately 10,800 pixels and to 48 spectral measurements, per acquisition date.

## Appendix A

**Table A2:** Bhattacharyya coefficient (B-coefficient) between distributions of vegetation indices, calculated from UAV and ground-based measurements, corresponding to each production system (i.e., non-mixed and mixed varieties). Only values calculated for the two last data acquisitions (84 and 99 days after planting—DAP) are presented. The index giving the best treatments distinction (i.e., largest B-coefficients) for both sensor systems is indicated in red.

Vegetation Indices	B-Coefficient				Difference between B-Coefficients (99 DAP–84 DAP)	
	84 DAP		99 DAP			
	UAV	Ground	UAV	Ground	UAV (Order)	Ground (Order)
NDVI	0.052	0.049	0.289	0.459	0.237 (6)	0.410 (7)
WDVI	0.008	0.003	0.258	0.564	0.250 (4)	0.561 (2)
OSAVI	0.014	0.011	0.262	0.558	0.248 (5)	0.547 (4)
MCARI	0.041	0.011	0.264	0.419	0.223 (8)	0.408 (8)
TCARI	0.037	0.007	0.268	0.444	0.231 (7)	0.437 (6)
MCARI/OSAVI	0.048	0.007	0.189	0.297	0.141 (9)	0.290 (10)
TCARI/OSAVI	0.029	0.004	0.053	0.184	0.024 (14)	0.180 (15)
MCARI_re_	0.009	0.007	0.285	0.535	0.276 (3)	0.528 (5)
MCARI/OSAVI_re_	0.010	0.007	0.302	0.559	0.292 (2)	0.552 (3)
TCARI/OSAVI_re_	0.018	0.004	0.089	0.189	0.071 (11)	0.185 (14)
CI_re_	0.006	0.008	0.127	0.319	0.121 (10)	0.311 (9)
CI_g_	0.009	0.000	0.023	0.228	0.014 (15)	0.228 (11)
MCARI2	0.024	0.011	0.333	0.594	0.309 (1)	0.583 (1)
REP	0.007	0.010	0.042	0.004	0.035 (13)	−0.006 (16)
MTCI	0.010	0.003	0.067	0.217	0.057 (12)	0.214 (12)
PRI	0.003	0.033	0.003	0.226	0.000 (16)	0.193 (13)
